# Long-term aspirin use for primary cancer prevention: An updated systematic review and subgroup meta-analysis of 29 randomized clinical trials

**DOI:** 10.7150/jca.49001

**Published:** 2020-09-14

**Authors:** Qibiao Wu, Xiaojun Yao, Hongwei Chen, Zhengtang Liu, Ting Li, Xingxing Fan, Guilin Zhang, Lili Yu, Min Chen, Cong Xu, Ruonan Zhang, Bi Chen, Xinbing Sui, Elaine Lai-Han Leung

**Affiliations:** 1 State Key Laboratory of Quality Research in Chinese Medicine, Macau University of Science and Technology, Macau, China.; 2 Faculty of Chinese Medicine; Macau University of Science and Technology, Macau, China.; 3 Faculty of Medicine; Macau University of Science and Technology, Macau, China.; 4 Department of Geriatrics, Xiyuan Hospital of China Academy of Chinese Medical Sciences, Beijing, P.R. China.; 5 Holistic Integrative Pharmacy Institutes and Department of Medical Oncology, The Affiliated Hospital of Hangzhou Normal University, College of Medicine, Hangzhou Normal University, Hangzhou, Zhejiang, China.; 6 Key Laboratory of Elemene Class Anti-cancer Chinese Medicine of Zhejiang Province and Engineering Laboratory of Development and Application of Chinese Medicine from Zhejiang Province, Hangzhou Normal University, Hangzhou, Zhejiang, China.

**Keywords:** long-term, aspirin, cancer, primary prevention, systematic review, subgroup meta-analysis, randomized clinical trials

## Abstract

**Background and objective:** Long-term aspirin use for the primary prevention of cancer remains controversial, and variations in the effect of aspirin use on cancer outcomes by aspirin dose, follow-up duration, or study population have never been systematically evaluated. The objective of this study was to evaluate the effect of aspirin on primary cancer prevention and to determine whether the effect differed according to aspirin dose, follow-up duration, or study population.

**Materials and methods:** Seven electronic databases were searched from inception to September 30, 2019. Randomized clinical trials (RCTs) that compared aspirin use versus no aspirin use in participants without pre-existing cancer and reported cancer outcomes were selected. Data were screened and extracted by different investigators. Analyses were performed using Review Manager 5.3 and Comprehensive Meta-Analysis 2.0. Total cancer incidence was defined as the primary clinical endpoint. Total cancer mortality, all-cause mortality, major bleeding, and total bleeding events were the secondary outcomes. Subgroup analyses were conducted based on aspirin dose, follow-up duration, and study populations.

**Results:** Twenty-nine RCTs that randomized 200,679 participants were included. Compared with no aspirin, aspirin use was not associated with significant reductions in total cancer incidence (RR = 1.01, 95% CI: 0.97 to 1.04, *P* = 0.72), total cancer mortality (RR = 1.00, 95% CI: 0.93 to 1.07, *P* = 0.90), or all-cause mortality (RR = 0.98, 95% CI: 0.94 to 1.02, *P* =0.31); however, aspirin use was associated with a 44% increase in the risk of major bleeding (RR = 1.44, 95% CI: 1.32 to 1.57, *P* < 0.00001) and a 52% increase in the risk of total bleeding events (RR = 1.52, 95% CI: 1.33 to 1.74, *P* < 0.00001). Subgroup analyses demonstrated consistent results.

**Conclusions:** Long-term aspirin use in individuals without pre-existing cancer was not associated with a significant reduction in total cancer incidence, cancer mortality, or all-cause mortality; however, aspirin use was associated with a significant increase in the risk of bleeding. Therefore, aspirin is not an appropriate choice for the primary cancer prevention.

## Introduction

There were approximately 18.1 million new cancer cases and 9.6 million cancer deaths worldwide in 2018 and the cancer incidence and deaths have been rapidly increasing 1-5. According to the WHO's report, 30-50% of cancer cases are preventable 6, but the methods for preventing cancer remain a major unanswered issue. There are some recognized prevention strategies, such as adopting healthy lifestyles, avoiding risk factors, etc. 6. However, there are also some controversial interventions for primary cancer prevention, such as aspirin use.

Over the last few decades, continuous long-term aspirin intake has been used as a chemopreventive approach for primary cancer prevention 7-9. Some studies have shown that this intervention reduced the morbidity and mortality rates of cancer 8-11; however, some other studies have found no overall association between them 12, 13. A few studies, including the ARRIVE and ASPREE trials, two high-quality randomized controlled trials (RCTs) published in 2018, demonstrated increased cancer incidence and mortality with aspirin use 14, 15. The results are conflicting, and the effect of aspirin on primary cancer prevention remains unclear and controversial.

A few previous meta-analyses have evaluated the role of aspirin use in primary cancer prevention, but most of them included observational trials or cohort studies which, compared with RCTs, might weaken the strength of the evidence 11, 12, 16-18. Some studies only focused on one certain type of cancer 16, 19, 20, one specific population, such as cardiovascular disease (CVD) prevention population 12, 21, 22, or the effect of low-dose of aspirin 23.

Aspirin's effect on primary cancer prevention has not been clearly established, and subgroup analyses based on aspirin dose, follow-up period, and study population have not been comprehensively conducted 12, 18. The U.S. Preventive Services Task Force (USPSTF) emphasized the need for more research into the effect of long-term aspirin use on the overall occurrence of cancer according to various aspirin doses and by subgroups, including patient characteristics, baseline cancer risk, comorbid conditions, etc. 12, 18.

This updated meta-analysis included all eligible RCTs to further evaluate the efficacy and safety of aspirin use for primary cancer prevention and to determine whether the effect differs according to aspirin dose, follow-up duration, or study population.

## Methods

We performed this systematic review and subgroup meta-analysis following the Preferred Reported Items for Systematic Review and Meta-analysis (PRISMA) guidelines 24. This study has been registered with the International Prospective Register of Systematic Reviews (PROSPERO): CRD42019134083. The methods used in this systematic review were described in the published protocol 25. Ethical approval was not required because all the materials were published studies.

### Data source

Two independent reviewers (QB Wu and HW Chen) performed a comprehensive search of the PubMed, Embase, ClinicalTrials.gov, Anzctr.org.au, Cochrane Library, Google Scholar and ScienceDirect databases without restriction on language or publication period. A conventional search was also performed to find potential studies that were not indexed in the electronic databases. Furthermore, the reference lists of all the related articles were reviewed to identify potential RCTs. The last search date was September 30, 2019. No trials were excluded due to their publication status or language.

### Study selection

All RCTs comparing aspirin versus no aspirin (defined as placebo or no treatment) and reporting cancer incidence and/or cancer deaths as outcomes were selected and assessed for inclusion in our research. The trials included in this study met the following criteria: (1) RCT study design; (2) participants without known preexisting cancer (primary prevention of cancer); (3) aspirin at any dose compared with no aspirin; (4) follow-up of at least 1 year; and (5) cancer incidence and/or cancer deaths reported as outcomes.

Exclusion criteria were as follows: (1) studies on secondary or tertiary prevention of cancer, treatment of cancer, cancer remission, cancer recurrence or cancer metastases; (2) studies in which the participants were nonhuman populations, pregnant women, institutionalized individuals or postsurgical patients; (3) studies of high-incidence familial cancer syndromes (e.g., Lynch syndrome, etc. ); (4) trials that were not RCTs; and (5) studies where the full-text article was unavailable or the data were unextractable.

All the candidate articles were screened by two independent investigators (QB Wu and HW Chen) on the basis of title and abstract. The full texts were retrieved for further evaluation according to the inclusion and exclusion criteria. All inclusion disagreements were resolved by consensus.

### Data extraction

Two investigators (QB Wu and XJ Yao) independently rated the included RCTs and extracted the data. An intention-to-treat (ITT) analysis was used to analyze the results whenever possible.

We summarized the characteristics of all included RCTs in Table 1 and performed a meta-analysis using Review Manager (RM) 5.3 (Copenhagen: The Nordic Cochrane Centre, The Cochrane Collaboration, 2014) and Comprehensive Meta-Analysis (CMA) 3.0 (Biostat, Englewood, NJ, United States; 2016) software to assess the effects of aspirin on cancer outcomes.

Two independent reviewers (QB Wu and HW Chen) appraised the risk of bias in the included trials using the Cochrane Risk of Bias Tool for Randomized Controlled Trials. 26 The following criteria were used to evaluate bias in each trial: random sequence generation; concealment of allocation; blinding of participants and personnel; blinding of outcome assessment; incomplete data; selective reporting; and other bias. The risk of bias was classified as 'low', 'high', or 'unclear'. The Jadad scale was also used to evaluate the quality of the included trials and a trial was considered high quality if the Jadad score was 3 or greater 27.

If there were disagreements, a third reviewer (ELH Leung) independently repeated the extraction, analysis, and interpretation of the data, and disagreements were solved by discussion until a consensus was reached.

### Outcomes

Total cancer incidence was defined as the primary clinical endpoint. Total cancer mortality, all-cause mortality, major bleeding, and total bleeding events were the secondary outcomes.

### Subgroup analysis

We performed subgroup analyses of total cancer incidence, total cancer mortality, all-cause mortality, and bleeding events based on aspirin dose, follow-up duration, and study populations.

### Data synthesis

All analyses were performed using RM 5.3, CMA 3.0 and Trial Sequential Analysis (TSA) software (Copenhagen Trial Unit, Centre for Clinical Intervention Research, Copenhagen, Denmark; 2011). Dichotomous data were summarized as risk ratios (RR) with 95% confidence intervals (CIs). Heterogeneity among the studies was assessed using the *I*-squared test. Substantial heterogeneity was indicated by *I*^2^ > 50%, and a random-effects model was used (Review Manager version 5, Cochrane Collaboration, Copenhagen, Denmark) to estimate the summary RR and 95% CI; otherwise, a fixed-effects model was applied 26, 28-33. If quantitative synthesis was not appropriate, a systematic narrative synthesis of the information was provided to summarize and explain the features and findings of the included RCTs 25, 34-36. The Grading of Recommendations Assessment, Development and Evaluation (GRADE) working group methodology was used to assess the strength of the body of evidence 37.

Egger's test and funnel plots were applied to examine the potential bias in the RCTs included in the meta-analysis when the number of RCTs was ≥ 10 38.

Subgroup analysis, sensitivity analysis, and Trial Sequential Analysis were applied to assess the robustness of the results and calculate the required sample size in the meta-analysis. 39 We also performed a meta-regression analysis to examine the potential heterogeneity and the impact of the moderator variables on the study effect size.

### Quality of evidence

The risk of bias for each included study was evaluated by two independent reviewers (QB Wu and XJ Yao) using the GRADE approach 37. Disagreements regarding a quality downgrade or upgrade were discussed with the third reviewer (XJ Yao) until a consensus was reached. The quality of the evidence was classified into four levels: “high”, “moderate”, “low” and “very low”. The quality of evidence was downgraded according to five domains: (I) limitation of the study design, (II) inconsistency, (III) indirectness, (IV) imprecision, (V) publication bias 37.

## Results

### Study search and study characteristics

As shown in Supplementary Figure S1, 1,369 records were identified through the literature search; 572 of them were duplicates. Reviews, letters, case reports, and basic research were removed after the titles and abstracts were read (n = 671). The full texts of 126 candidate papers were then evaluated, and 97 were removed for the following reasons: duplicated data reported (n = 38), nonrandomized controlled study design (n = 35), insufficient data (n = 18), and familial cancer syndromes (n = 6). In total, 29 trials met the inclusion criteria 14, 15, 40-69.

These 29 RCTs, which comprised 200,679 individuals, were included (Supplementary Figure S1 and Table 1) 14, 15, 40-69. The included RCTs were performed and published from 1976 to 2018. The number of participants in each RCT varied from 475 to 39,876. The mean (or median) age of the participants ranged from 44 to 74 years in the different trials. All the included RCTs compared aspirin to placebo or no treatment. Sixteen trials used a daily aspirin dose ≤ 100 mg for the participants, four trials used a dose of 100-300 mg/d (one trial used 325 mg every other day, and 162.5 mg/d was regarded as the daily dose), six trials used >300 mg/d, one trial used 81 mg/d or 325 mg/d in different groups, one used 300 mg/d or 1200 mg/d in different groups, 68 and one did not clearly report the daily dose of aspirin 62. The mean follow-up ranged from 1.8 to 12 years. The characteristics of the 29 trials are shown in Table 1.

### Risk of bias and methodologic quality

The risk of bias and methodologic quality of all the included RCTs were evaluated and are presented in Table 1, Supplementary Table S1 and Supplementary Figure S2, S3. The methodologic quality of all the included RCTs was high; twenty-five trials scored 5 out of 5 for methodological quality (Jadad score), and four trials scored 3 out of 5 (Tables 1). The randomization methods were distinctly reported in all trials. Twenty-five trials were double-blinded. Four trials were open-label and end-point blinded; allocation concealment and blinding of the participants and personnel contributed to a high risk of bias in these four trials 45, 53, 56, 61. In all included trials, the data were complete. The presence of any other bias was not clear (Supplementary Table S1 and Supplementary Figure S2, S3).

### Outcome measures

The findings of the meta-analyses are summarized in Table 2.

### Total cancer incidence, cancer mortality and all-cause mortality

**Figure 1:**
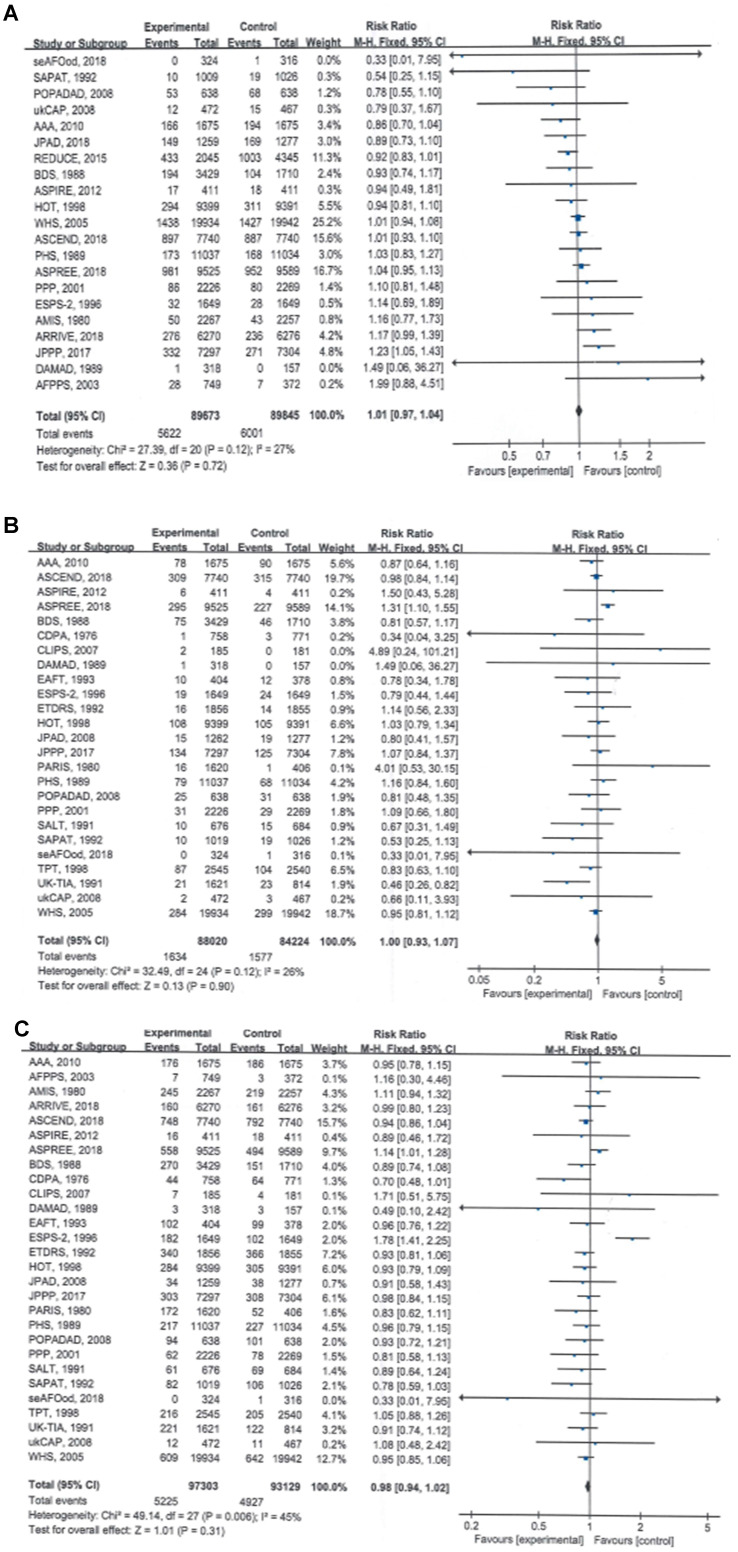
Forest plots showing that long-term aspirin use was not associated with significant reductions in total cancer incidence, total cancer mortality or all-cause mortality. A) Total cancer incidence, B) total cancer mortality, C) all-cause mortality.

Twenty-one of the included RCTs involving 179,518 participants observed and reported total cancer incidence, the primary clinical endpoint of this study; the pooled data showed that compared with no aspirin, aspirin use was not associated with a significant reduction in total cancer incidence (RR = 1.01, 95% CI: 0.97 to 1.04, *P* = 0.72) (Table 2; Figure 1A).

The results of the meta-analyses also showed that the total cancer mortality rate (RR = 1.00, 95% CI: 0.93 to 1.07, *P* = 0.90) and all-cause mortality rate (RR = 0.98, 95% CI: 0.94 to 1.02, *P* = 0.31) were not significantly different between the aspirin and no-aspirin groups (Table 2; Figure 1B and 1C).

No significant heterogeneity was found for any of the three outcomes (*I*² = 27%, 26% and 45%, respectively), and the fixed-effects model was used to pool the trial results.

### Bleeding events

**Figure 2:**
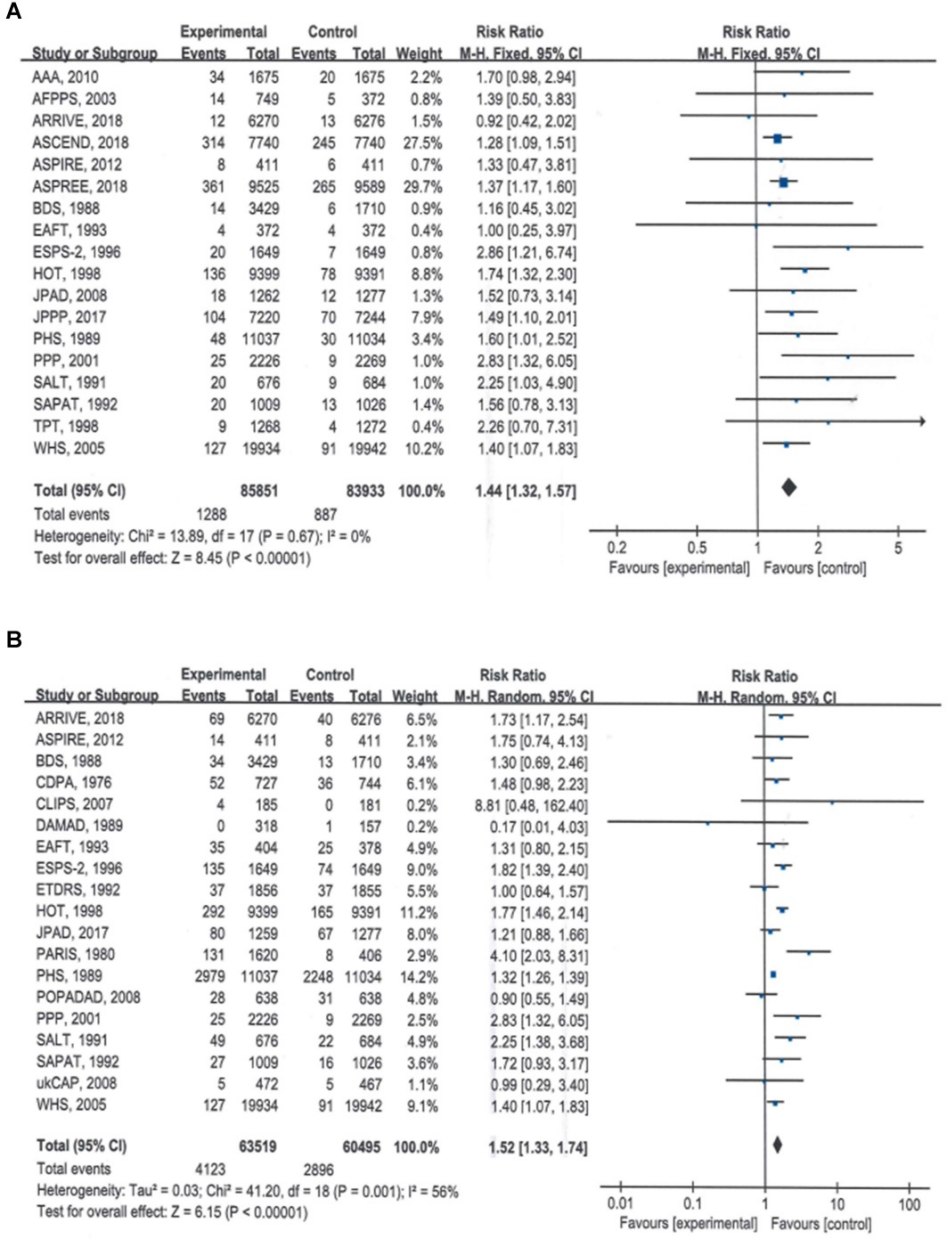
Forest plots showing that the risks of major bleeding and all bleeding events were significantly higher with aspirin than with placebo. A) Major bleeding events, B) total bleeding events.

The summary estimates indicated that compared with no aspirin, aspirin use significantly increased the risk of major bleeding by 44% (RR = 1.44, 95% CI: 1.32 to 1.56, *P* < 0.00001) (Table 2; Figure 2A) and the risk if total bleeding events by 52% (RR = 1.52, 95% CI: 1.33 to 1.74, *P* < 0.00001) (Table 2; Figure 2B).

### Subgroup analyses

#### Subgroup analyses of total cancer incidence, cancer mortality and all-cause mortality based on aspirin dose

The results of the subgroup analyses based on aspirin dose showed that different daily dose of aspirin were not associated with a significant reduction in total cancer incidence [≤ 100 mg (RR = 1.02, 95% CI: 0.98 to 1.06, *P* = 0.31); 100 - 300 mg (RR = 1.01, 95% CI: 0.82 to 1.24, *P* = 0.92); > 300 mg (RR = 1.01, 95% CI: 0.83 to 1.23, *P* = 0.91)] (Table 2; Supplementary Figure S4A), total cancer mortality [≤ 100 mg (RR = 1.01, 95% CI: 0.94 to 1.08, *P* = 0.85); 100-300 mg (RR = 1.04, 95% CI: 0.80 to 1.35, *P* = 0.76); > 300 mg (RR = 0.91, 95% CI: 0.68 to 1.22, *P* = 0.53)] (Table 2; Supplementary Figure S4B), or all-cause mortality [≤ 100 mg (RR = 0.97, 95% CI: 0.93 to 1.01, *P* = 0.16); 100-300 mg (RR = 0.94, 95% CI: 0.83 to 1.07, *P* = 0.36); > 300 mg (RR = 0.94, 95% CI: 0.86 to 1.01, *P* = 0.11)] (Table 2; Supplementary Figure S4C).

The meta-regression analysis showed that total cancer incidence, cancer mortality or all-cause mortality did not vary significantly with respect to daily dose of aspirin (from ≤100 mg to >300 mg) (Supplementary Figure S5).

#### Subgroup analyses of total cancer incidence, cancer mortality and all-cause mortality based on follow-up duration

There was no significant reduction in total cancer incidence with aspirin use when different follow-up durations were evaluated [1-5 years (RR = 0.99, 95% CI: 0.93 to 1.05,* P* = 0.65); 5-10 years (RR = 1.01, 95% CI: 0.90 to 1.14, *P* = 0.82); >10 years (RR = 1.00, 95% CI: 0.94 to 1.06,* P* = 0.96)] (Table 2; Supplementary Figure S6A).

For total cancer mortality or all-cause mortality, the stratified meta-analysis showed similar results; aspirin use was not associated with either total cancer mortality [1-5 years (RR = 1.08, 95% CI: 0.96 to 1.22,* P* = 0.20); 5-10 years (RR = 0.92, 95% CI: 0.84 to 1.01,* P* = 0.10); >10 years (RR = 1.00, 95% CI: (0.88 to 1.14,* P* = 1.00)] (Table 2; Supplementary Figure S6B) or all-cause mortality [1-5 years (RR = 0.97, 95% CI: 0.88 to 1.08,* P* = 0.63); 5-10 years (RR = 0.96, 95% CI: 0.90 to 1.02,* P* = 0.18); or >10 years (RR = 0.95, 95% CI: 0.87 to 1.04,* P* = 0.29)] (Table 2; Supplementary Figure S6C).

The meta-regression analysis indicated that total cancer incidence, cancer mortality or all-cause mortality did not vary significantly with respect to follow-up duration (from 1-5 years to >10 years) (Supplementary Figure S7).

A subgroup analysis was also conducted by only including the RCTs that used an aspirin dose ≤ 100 mg/d for > 5 years. This analysis showed that using a low dose of aspirin (≤ 100 mg/d) for more than five years did not result in a lower total cancer incidence (RR = 1.01, 95% CI: 0.93 to 1.10,* P* = 0.78), total cancer mortality (RR = 0.96, 95% CI: 0.87 to 1.04,* P* = 0.24) or all-cause mortality (RR = 0.96, 95% CI: 0.91 to 1.02,* P* = 0.16) (Table 2; Supplementary Figure S8).

#### Subgroup analyses of total cancer incidence, cancer mortality and all-cause mortality based on study population

Aspirin did not decrease the total cancer incidence in the different subgroups of participants, including the healthy population (RR = 1.02, 95% CI: 0.97 to 1.07, *P* = 0.54), patients with diabetes mellitus (RR = 0.95, 95% CI: 0.84 to 1.08, *P* = 0.42), participants with CVD or at increased risk of CVD (RR = 1.04, 95% CI: 0.92 to 1.19, *P* = 0.50), individuals at increased risk of cancer (RR = 1.00, 95% CI: 0.66 to 1.54, *P* = 0.99), or patients with peripheral arterial disease or venous thromboembolism (RR = 0.81, 95% CI: 0.60 to 1.10, *P* = 0.18) (Table 2; Supplementary Figure S9A).

Subgroup analyses also showed that the risks of cancer mortality or all-cause mortality in the above subgroups were not reduced by long-term aspirin use (all* P* > 0.05) (Table 2; Supplementary Figure S9B and S9C).

#### Subgroup analyses of bleeding events based on aspirin dose

The summary estimates indicated that compared with no aspirin, all three different daily doses of aspirin significantly increased the risk of major bleeding [≤ 100 mg (RR = 1.44, 95% CI: 1.32 to 1.57, *P*< 0.00001), 100-300 mg (RR = 1.58, 95% CI: 1.03 to 2.42, *P* = 0.04), or > 300 mg (RR = 1.49, 95% CI: 1.02 to 2.18, *P* = 0.04)] (Table 2; Figure S10A).

For total bleeding events, the results were similar, and the risk was significantly increased in the three subgroups treated with different daily doses of aspirin [≤ 100 mg (RR = 1.61, 95% CI: 1.37 to 1.89, *P* < 0.00001); 100-300 mg (RR = 1.45, 95% CI: 1.13 to 1.85, *P* = 0.003); or > 300 mg (RR = 1.72, 95% CI: 1.06 to 2.78, *P* = 0.03)] (Table 2; Figure S10B).

#### Subgroup analyses of bleeding events based on follow-up duration

Subgroup analyses based on follow-up duration showed that the risk of major bleeding and total bleeding events significantly increased after three different follow-up durations (all *P* < 0.05) (Table 2; Supplementary Table 2 and Supplementary Figure S11A, S11B).

### Sensitivity analyses and trial sequential analysis

Generally, there was good homogeneity among the included clinical trials. In particular, the above subgroup analysis results based on the daily dose of aspirin, follow-up duration, and study populations confirmed the robustness of the findings.

With regard to cancer incidence, the primary outcome, the pooled data showed that aspirin use did not significantly decrease the total cancer incidence. The results were similar when the sensitivity analyses were based on study quality (when only double-blind RCTs were selected) (RR = 1.00, 95% CI: 0.96 to 1.04, *P* = 0.96), study sample size (≥ 2,000 subjects in each group) (RR = 1.03, 95% CI: 0.99 to 1.07, *P* = 0.10), and publication year (studies published since the year 2000) (RR = 1.01, 95% CI: 0.97 to 1.05, *P* = 0.55) and when studies that enrolled participants with increased risk of cancer were excluded (RR = 1.00, 95% CI: 0.97 to 1.04, *P* = 0.31) (Supplementary Table 3).

Trial sequential analysis indicated that aspirin was not significantly superior to no aspirin, and the cumulative sample size of all the RCTs reached the required information size (RIS) needed for a conclusive and reliable meta-analysis (Supplementary Figure S12), suggesting that the findings of the meta-analysis were robust for the total cancer incidence outcome. The meta-regression analysis showed that the total cancer incidence did not vary significantly with respect to daily dose of aspirin (from ≤100 mg to >300 mg) [LogOR = 0.0215 - 0.0025 daily dose, (u = 0.31, *P* = 0.96)], or follow-up duration (from 1-5 years to >10 years) [LogOR = 0.0057 - 0.0043 Follow-up duration, u = 0.11, *P* = 0.91] (Supplementary Figure S5A, S7A).

For the total cancer mortality, all-cause mortality, major bleeding, and total bleeding events, the sensitivity and subgroup analyses showed similar results.

### Quality of evidence and publication bias

In the 29 included RCTs, 25 were double-blinded trials with overall low methodological bias risk. All available RCTs had large sample sizes, from 475 to 39,876 individuals. For the primary outcome and most of the secondary outcomes, the results had good robustness. Heterogeneity was present in a minority (7/53) of the outcomes, and the quality of evidence was downgraded by one level (total bleeding events, total cancer incidence after a follow-up of 5-10 years and in populations at increased risk of CVD, etc.). According to the GRADE guidelines, the quality of evidence for the outcomes measured was moderate to high, and majority were of high quality (Table 2; Supplementary Table S 2).

There was no evidence of publication bias for total cancer incidence, the primary outcome (Egger's test *P* = 0.348) (Supplementary Figure S13).

## Discussion

The results of previous pooled analyses and meta-analyses of studies of long-term aspirin use for the primary prevention of cancer were inconsistent; most of them showed that aspirin had a substantial net benefit for cancer primary prevention 8, 11, 17, 70, but a few demonstrated that aspirin was not associated with a reduction in the cancer outcomes 12, 18, 71. The discrepancies in the results might be caused by the varied inclusion criteria used in the different analyses. Most of the previous meta-analysis included observational and/or cohort studies 11, 12, 16-18, which undermined the strength of the evidence regarding the association between aspirin use and cancer incidence or mortality.

Evidence from good-quality meta-analyses of RCTs is at the top of the evidence hierarchy, but there were very limited meta-analyses of RCTs evaluating the effect of long-term aspirin use on cancer incidence or mortality, and almost all of them only included a primary CVD prevention population 12, 13, 22. A recent meta-analysis of RCTs assessed the overall effect of aspirin on cancer outcomes 71; however, many eligible RCTs, including some new trials such as ARRIVE, JPPP, etc., were not included 14, 15. In addition, two included trials were duplicated 53, 55, which might have weakened the strength of the evidence of the meta-analysis 72. Therefore, it was necessary to conduct an updated systematic review of all eligible RCTs to further evaluate the overall effect of long-term aspirin use on cancer outcomes.

In our study, 29 eligible RCTs that randomized 200,679 participants were included. All RCTs comparing aspirin use to no aspirin use in participants without pre-existing cancer that reported cancer outcomes were selected. To the best of our knowledge, our meta-analysis included the largest number of relevant RCTs and participants, and it is the first comprehensive subgroup meta-analysis of long-term aspirin use for cancer primary prevention based on aspirin dose, follow-up duration, and study populations, which are considered potential modifiers of the effects of aspirin on cancer outcomes. Both the USPSTF and a UK panel called for more research into the effect of long-term aspirin use on cancer primary prevention according to a range of doses and by subgroups, including baseline cancer risk, or comorbid conditions, etc.^12^, ^13^, ^18^, ^73^ Though the existing research in this field is far from enough, the findings of the present study may add some evidence regarding the variation in the effects of aspirin use on cancer outcomes by aspirin dose, follow-up duration, or different populations.

### Effect of aspirin on total cancer incidence, cancer mortality, and all-cause mortality

Our data indicated that, compared with no aspirin, long-term aspirin use did not result in a significantly lower risk of total cancer incidence (*P* = 0.75), cancer mortality (*P* = 0.81), or all-cause mortality (*P* = 0.27). The results clearly demonstrated that the current practice of prescribing aspirin as a chemopreventive agent for the primary prevention of cancer brought no benefit to the individuals who underwent aspirin therapy. According to the GRADE guidelines, the quality of evidence for the cancer outcomes (total cancer incidence and mortality) in our study was high.

Trial sequential analysis of total cancer incidence, the primary endpoint, indicated that the use of aspirin in the experimental group was not superior to the intervention (no aspirin) in the control group and that the cumulative sample size of all included RCTs reached the required size for a conclusive and reliable meta-analysis.

### Association between aspirin dose, follow-up duration and cancer outcomes

Many studies have shown that long-term aspirin use (especially low-dose aspirin use) reduced the risk of developing and dying from cancer and that the benefit increased with the duration of treatment 8, 9, 70, 74. A pooled analysis of six CVD primary prevention studies indicated that daily low-dose aspirin reduced the risk of cancer and that the effect was greater for those who received treatment for at least 5 years 8. A previous meta-analysis including 218 observational studies found that taking a daily low-dose of 75-100 mg for at least five years dramatically reduced the risks of cancer morbidity and mortality 11.

In our study, subgroup analyses based on aspirin dose or follow-up duration showed that different daily doses of aspirin (≤ 100 mg, 100-300 mg, or > 300 mg) and different follow-up durations (1-5 years, 5-10 years, or >10 years) were not associated with a significant reduction in total cancer incidence, cancer mortality or all-cause mortality. The aspirin dose or follow-up duration did not show any impact on the effect of aspirin, which was not greater for those who received low-dose aspirin or who underwent treatment for more than 5 years.

We also performed a subgroup analysis by only including the participants who used low-dose (≤100 mg/d) aspirin for more than five years. The results showed no significant reduction in total cancer incidence (*P* = 0.78), total cancer mortality (*P* = 0.33) or all-cause mortality (*P* = 0.16) with aspirin use. The results of the meta-regression analyses confirmed the above findings of the subgroup analyses. Several previous studies reported that daily use of low-dose aspirin for at least five years reduced the risk of cancer and cancer mortality 8, 11, 74, but they were not meta-analyses of RCTs. Our findings differed from theirs.

### Aspirin use for cancer primary prevention in different study populations

Our study stratified the participants by health status and baseline risk of CVD, cancer, or comorbid conditions, etc., to evaluate the impact of the effect of aspirin on different populations 12, 21, 22, 71. Subgroup analyses based on population showed that the risks of total cancer incidence, cancer mortality or all-cause mortality were not reduced by aspirin use in five different subgroups, including the healthy population, patients with diabetes mellitus, participants with CVD or at increased risk of CVD, individuals with increased risk of cancer, or patients with peripheral arterial disease or venous thromboembolism.

### The risk of bleeding events

Toxic effects are very common in individuals treated with long-term aspirin; bleeding events are the leading side effects. The present meta-analysis showed that long-term aspirin use was associated with a significant increase in the risk of major bleeding and total bleeding events. Even in the individuals who used low-dose aspirin (≤ 100 mg) for a relatively short duration (1-5 years), the bleeding risk was still significantly increased.

### Long-term aspirin use for the prevention of specific cancers

Our data indicated that long-term aspirin use as a primary cancer prevention measure had no benefit; however, aspirin use was associated with a significantly increased bleeding risk. Therefore, the present evidence does not favor the use of aspirin as a primary prevention strategy in the general population or in the above subgroups.

Because we excluded specific populations with familial cancer syndromes (Lynch syndrome, etc.) 75, more studies are needed to evaluate the benefit and risk of aspirin as an anticancer intervention for these populations.

In this study, we only evaluated the effect of long-term aspirin use for overall cancer prevention. We did not evaluate the effect of this intervention on the prevention of specific subtypes of cancer. There seem to be some evidence to support the use of aspirin for the chemoprevention of a few specific cancers, especially colorectal cancer 22, 76; more research is needed to further assess the effect of aspirin use on different cancers 21, 77-82.

## Limitations

Our study had some limitations: firstly, although the included trials collected data on cancer outcomes, most of them were designed as RCTs to evaluate aspirin's effect on the cardiovascular system or on non-cancer outcomes (outcomes other than primary cancer prevention). Therefore, some potential confounding factors could have affected the outcomes, thus masking the actual association or falsely demonstrating an association between the aspirin treatment and cancer outcomes.

Second, the cancer rates were much lower than in the setting of CVD or other comorbidities. The sample size needed for primary cancer prevention trials is larger, and the study duration must be longer; the sample size or follow-up duration of some of the included RCTs might have been insufficient.

Third, some of the included trials had heterogeneity and potential risk of bias, and the quality of evidence of some outcomes was moderate, thus weakening the trustworthiness and strength of evidence from this systematic review.

Last, individual patient data were not sufficient, and consequently, information for more stratified analyses (e.g., by age, sex, risk factors, cancer type) was limited.

## Conclusions

From the available evidence, our data indicated that compared with no aspirin, the long-term use of aspirin in individuals without pre-existing cancer was not associated with a reduction in total cancer incidence, cancer mortality, or all-cause mortality; however, aspirin use was associated with a significant increase in the risk of bleeding in this population. Therefore, aspirin might not be an appropriate choice for the primary prevention of cancer. Prospective RCTs of the role of aspirin in primary cancer prevention are warranted.

## Supplementary Material

Supplementary figures and tables.

## Figures and Tables

**Table 1:** Principal characteristics of the studies included in the meta-analysis

Source	Trial design	Jadad score	Country	Study population	Total randomized	Average daily dose of aspirin (mg)	Comparator	Mean follow-up	Outcomes
AAA, 2010 40	RCT, double-blind	5	UK	Aged 50-75 y with ankle brachial index ≤0.95	3350	100 daily	Placebo	8.2	1, 2,3,4
AFPPS, 2003 41	RCT, double-blind	5	US	Individuals with a history of colorectal adenoma	1121	81 or 325 daily	Placebo	2.7	1, 3,4
AMIS, 1980 42	RCT, double-blind	5	US	Aged 30-69 y with prior myocardial infarction.	4524	1000 daily	Placebo	3	1, 3
ARRIVE, 2018 14	RCT, double-blind	5	Germany, Italy, Ireland, Poland, Spain, UK, US.	Males at increased risk of CVD	12546	100 daily	Placebo	6	1, 2,3,4
ASCEND, 2018 43	RCT, double-blind	5	UK	Individuals with diabetes, aged ≥40 y	15480	100 daily	Placebo	7.4	1, 2,3,4
ASPIRE, 2012 44	Multi-center, RCT, double-blind	5	Australia, India, New Zealand, Singapore, Argentina.	Patients with unprovoked venous thromboembolism	822	100 daily	Placebo	3.1	2,3,4
ASPREE, 2018 15	RCT, double-blind	5	Australia and US.	The healthy elderly	19114	100 daily	Placebo	4.7	1, 2,3,4
BDS, 1988 45	RCT, open-label, Endpoint blind	3	UK	Male physicians who were apparently healthy.	5139	500 daily	No aspirin	6	2,3,4
CDPA, 1980 46	RCT, double-blind	5	US	Men with prior myocardial infarction.	1529	972 daily	Placebo	1.8	2,3,4
CLIPS, 2007 47	RCT, double-blind	5	Europe	Patients with peripheral arterial disease	366	100 daily	Placebo	2	2,3,4
DAMAD, 1989 48	RCT, double-blind	5	France, UK	Aged 17-67 years, type I or II DM; early diabetic retinopathy	475	990 daily	placebo	3	1, 2,3
EAFT, 1993 49	RCT, double-blind	5	Europe (12 countries), Israel.	Aged > 25 years, with prior TIA or minor ischemic stroke	782	300 daily	No aspirin	2.3	2,3,4
ESPS-2, 1996 50	Multicenter, RCT, double-blind	5	Europe (13 countries).	Aged ≥ 18 years; with prior TIA or stroke	6602	50 daily	No aspirin	2	1, 2,3,4
ETDRS, 1992 51	Multicenter, RCT double-blind trial	5	US	Aged 18-70 years; with DM and diabetic retinopathy	3711	650 daily	No aspirin	5	2,3,4
HOT, 1998 52	RCT, double-blind	5	26 countries across Europe, North and South America, Asia.	Individuals with hypertension aged 50-80 y	18790	75 daily	Placebo	3.8	1,2, 3,4
JPAD, 2018 53/ 2017 54/2018 55	RCT, open-label, blinded end-point	3	Japan	Individuals with diabetes aged 30-85 y	2539	81 or 100 daily	No aspirin	4.37/10.7	1, 2,3,4
JPPP, 2018 56	RCT, open label, blind endpoint	3	Japan	Individuals aged 60-85y, with hypertension, dyslipidemia, or diabetes	14658	100 daily	No aspirin	6.5	1, 2,3,4
PARIS, 1980 57	RCT, double-blind	5	US, UK	Males and females with prior MI	2026	972 daily	Placebo	3.4	2,3,4
PHS, 1989 58/ 1998 59	RCT, double-blind	5	US	Disease-free male physicians aged 40-84 y	22071	162.5 daily (325 qod)	Placebo	5/12	1, 2,3,4
POPADAD, 2008 60	RCT, double-blind	5	UK	Individuals with diabetes, ABPI ≤0.99, aged ≥40 y	1276	100 daily	Placebo	6.7	1,2,3,4
PPP, 2001 61	RCT, open-label, blind endpoint	3	Italy	Individuals with ≥1 CVD risk factor	4495	100 daily	No aspirin	3.6	1, 2,3,4
REDUCE, 2015 62	Multicenter, RCT, double-blind	5	Europe, Canada, US, Puerto Rico.	Men at increased risk of prostate cancer, aged 48-77 y	6390	Unknown	Placebo	4	1, 3,4
SALT, 1991 63	RCT, double-blind	5	Sweden	Males and females with prior TIA or stroke	1360	75 daily	Placebo	2.7	1, 2,3,4
SAPAT, 1992 64	RCT, double-blind	5	Sweden	Aged 30-80 years with chronic stable angina pectoris	2035	75 daily	No aspirin	4.2	1, 3,4
seAFOod, 2018 65	Multicenter, RCT, double-blind	5	UK	Aged 55-73 years, at high risk in the English Bowel Cancer Screening Programme	709	300 daily	Placebo	5	1, 2,3
TPT, 1998 66	RCT, double-blind	5	UK	Men aged 45-69 y at high risk of CVD	5499	75 daily	No aspirin	6.8	2,3,4
ukCAP, 2008 67	RCT, double-blind	5	UK, Denmark.	Aged < 75 years, had colorectal adenoma removed	945	300 daily	No aspirin	3.4	1, 2,3
UK-TIA, 1991 68	RCT, double-blind	5	UK	Males and females with prior TIA or stroke	2449	300 or 1200 daily	Placebo	4	2,3,4
WHS, 2005 69	RCT, double-blind	5	US	Female health professionals ≥ 45 y	39876	50 daily (100 qod)	Placebo	10.1	1, 2,3,4

Abbreviations: **AAA**, Aspirin for Asymptomatic AtherosclerosisTrial; **AFPPS**, The Aspirin/Folate Polyp Prevention Study; **AMIS**, Aspirin Myocardial Infarction Study; **ARRIVE**, Aspirin to Reduce Risk of Initial Vascular Events; **ASCEND**, A Study of Cardiovascular Events in Diabetes; **ASPIRE**, Aspirin to Prevent Recurrent Venous Thromboembolism trial; **ASPREE**, Aspirin in Reducing Events in the Elderly; **BDS**, British Doctors Study; **CDPA**, Coronary Drug Project Research; **CLIPS**, Critical Leg Ischaemia Prevention Study; **CVD**, cardiovascular diseases;** DAMAD**, the Dipyridamole Aspirin Microangiopathy of Diabetes study;** DM**, diabetes mellitus; **EAFT**, European atrial fibrillation trial; **ESPS-2**, European Stroke Prevention Study 2; **ETDRS**, Early Treatment Diabetic Retinopathy Study; HOT, Hypertension Optimal Treatment; **JPAD**, Japanese Primary Prevention of Atherosclerosis with Aspirin for Diabetes; **JPPP**, Japanese Primary Prevention Project; **PARIS**, The Persantine-Aspirin Reinfarction Study; **PHS**, Physicians'Health Study; **POPADAD**, Prevention of Arterial Disease and Diabetes; **PPP**, Primary Prevention Project; **RCT**, RCT controlled trial; **T/C**, treatment group/control group; **REDUCE**, the Reduction by Dutasteride of Prostate Cancer Events study; **SAPAT**, The Swedish Angina Pectoris Aspirin Trial; **SALT**, Swedish Aspirin Low-dose Trial; **seAFOod**, The Systematic Evaluation of Aspirin and Fish Oil Polyp Prevention Trial; **TIA**, transient ischemic attack,** TPT**, Thrombosis Prevention Trial;** UK**, the United Kingdom; **US**, the United States; **ukCAP**, The United Kingdom Colorectal Adenoma Prevention; **UK-TIA**, The United Kingdom transient ischaemic attack; **WHS**, Women's Health Study. Some of the data for the AAA, HOT, BDS, and PHS trials were extracted from previous meta-analyses 8, 12, 18; 1.Total cancer incidence; 2. Total cancer mortality; 3. All-cause mortality; 4. Bleeding outcome.

**Table 2:** Summary of findings: long-term aspirin use for cancer primary prevention

Outcomes	No. of Studies	Events/no. of patients		*I* ^2^	Statistical method	Relative risk (95% CI)	*P* value	Quality of the evidence (GRADE)
		Aspirin	No Aspirin					
Total cancer incidence	21	5622/89673	6001/89845	27	RR (fixed), 95% CI	1.01 (0.97 to 1.04)	0.72	⊕⊕⊕⊕ High
Total cancer mortality	25	1634/88020	1577/84224	26	RR (fixed), 95% CI	1.00 (0.93 to 1.07)	0.90	⊕⊕⊕⊕ High
All-cause mortality	28	5225/97303	4927/93129	45	RR (fixed), 95% CI	0.98 (0.94 to 1.02)	0.31	⊕⊕⊕⊕ High
Major bleeding events	18	1288/85851	887/83933	0	RR (fixed), 95% CI	1.44 (1.32 to 1.57)	<0.00001*	⊕⊕⊕⊕ High
Total bleeding events	19	4123/63519	2896/60495	56	RR (random), 95% CI	1.52 (1.33 to 1.74)	<0.00001*	⊕⊕⊕Ο Moderate
**Subgroup analyses**								
***Total cancer incidence***								
*Dose of aspirin*								
≤100 mg/d	15	4920/80446	4835/80593	38	RR (fixed), 95% CI	1.02 (0.98 to 1.06)	0.31	⊕⊕⊕⊕ High
100-300 mg/d	2	185/11509	183/11501	0	RR (fixed), 95% CI	1.01 (0.82 to 1.24)	0.92	⊕⊕⊕⊕ High
>300 mg/d	3	256/6068	154/4349	9	RR (fixed), 95% CI	1.01 (0.83 to 1.23)	0.91	⊕⊕⊕⊕ High
*Follow-up duration*								
1-5 years	12	1944/30394	2477/32249	3	RR (random), 95% CI	0.99 (0.93 to 1.05)	0.65	⊕⊕⊕⊕ High
5-10 years	6	1918/27049	1706/25343	64	RR (random), 95% CI	1.01 (0.90 to 1.14)	0.82	⊕⊕⊕Ο Moderate
>10 years	3	1760/32230	1764/32253	0	RR (random), 95% CI	1.00 (0.94 to 1.06)	0.96	⊕⊕⊕⊕ High
*Study population*								
Healthy population	4	2786/43925	2651/42275	0	RR (random), 95% CI	1.02 (0.97 to 1.07)	0.54	⊕⊕⊕⊕ High
With DM	3	1099/9637	1124/9655	33	RR (random), 95% CI	0.95 (0.84 to 1.08)	0.42	⊕⊕⊕⊕ High
With CVD or at increased risk of CVD	8	1246/31972	1182/31847	52	RR (random), 95% CI	1.04 (0.92 to 1.19)	0.50	⊕⊕⊕Ο Moderate
At increased risk of cancer	3	326/2589	775/4008	48	RR (random), 95% CI	1.00 (0.66 to 1.54)	0.99	⊕⊕⊕⊕ High
With peripheral arterial disease or venous thromboembolism	2	70/1049	86/1049	0	RR (random), 95% CI	0.81 (0.60 to 1.10)	0.18	⊕⊕⊕⊕ High
***Total cancer mortality***								
*Dose of aspirin*								
≤100 mg/d	15	1413/66181	1406/66316	29	RR (fixed), 95% CI	1.01 (0.94 to 1.08)	0.85	⊕⊕⊕⊕ High
100-300 mg/d	5	112/12895	107/12869	0	RR (fixed), 95% CI	1.04 (0.80 to 1.35)	0.76	⊕⊕⊕⊕ High
>300 mg/d	6	120/8796	77/5713	0	RR (fixed), 95% CI	0.91 (0.68 to 1.22)	0.53	⊕⊕⊕⊕ High
*Follow-up duration*								
1-5 years	17	563/33416	499/31501	37	RR (fixed), 95% CI	1.08 (0.96 to 1.22)	0.20	⊕⊕⊕⊕ High
5-10 years	6	858/35961	885/34245	0	RR (fixed), 95% CI	0.92 (0.84 to 1.01)	0.10	⊕⊕⊕⊕ High
>10 years	3	426/32230	427/32253	0	RR (fixed), 95% CI	1.00 (0.88 to 1.14)	1.00	⊕⊕⊕⊕ High
*Study population*								
Healthy population	4	733/43925	640/42275	69	RR (random), 95% CI	1.06 (0.86 to 1.31)	0.58	⊕⊕⊕Ο Moderate
With DM	5	366/11814	379/11667	0	RR (random), 95% CI	0.96 (0.84 to 1.11)	0.61	⊕⊕⊕⊕ High
With CVD or at increased risk of CVD	12	525/30889	550/28907	24	RR (random), 95% CI	0.88 (0.76 to 1.03)	0.10	⊕⊕⊕⊕ High
At increased risk of cancer	2	2/648	4/643	0	RR (random), 95% CI	0.56 (0.12 to 2.66)	0.47	⊕⊕⊕⊕ High
With peripheral arterial disease or venous thromboembolism	3	32/1140	35/1139	0	RR (random), 95% CI	0.91 (0.56 to 1.45)	0.68	⊕⊕⊕⊕ High
***All-cause mortality***								
*Dose of aspirin*								
≤100 mg/d	18	3997/85515	4127/85652	0	RR (fixed), 95% CI	0.97 (0.93 to 1.01)	0.16	⊕⊕⊕⊕ High
100-300 mg/d	5	440/13043	460/13009	0	RR (fixed), 95% CI	0.94 (0.83 to 1.07)	0.36	⊕⊕⊕⊕ High
>300 mg/d	8	1190/11435	980/8342	13	RR (fixed), 95% CI	0.94 (0.86 to 1.01)	0.11	⊕⊕⊕⊕ High
*Follow-up duration*								
1-5 years	19	2432/36741	2154/34270	60	RR (random), 95% CI	0.97 (0.88 to 1.08)	0.63	⊕⊕⊕Ο Moderate
5-10 years	7	1967/29594	1904/27883	0	RR (random), 95% CI	0.96 (0.90 to 1.02)	0.18	⊕⊕⊕⊕ High
>10 years	2	826/30971	869/30976	0	RR (random), 95% CI	0.95 (0.87 to 1.04)	0.29	⊕⊕⊕⊕ High
*Study population*								
Healthy population	5	1830/45600	1700/43950	46	RR (fixed), 95% CI	1.00 (0.93 to 1.06)	0.90	⊕⊕⊕⊕ High
With DM	5	1219/11811	1300/11667	0	RR (fixed), 95% CI	0.94 (0.87 to 1.01)	0.08	⊕⊕⊕⊕ High
With CVD or at increased risk of CVD	11	2131/37760	2067/36570	0	RR (fixed), 95% CI	0.96 (0.91 to 1.02)	0.22	⊕⊕⊕⊕ High
At increased risk of cancer	2	19/1221	14/839	0	RR (fixed), 95% CI	1.10 (0.55 to 2.20)	0.79	⊕⊕⊕⊕ High
With peripheral arterial disease or venous thromboembolism	2	23/596	22/592	0	RR (fixed), 95% CI	1.04 (0.59 to 1.85)	0.89	⊕⊕⊕⊕ High
**Dose of aspirin and follow-up duration**								
***Aspirin ≤100 mg/d*** ***for more than five years***								
Total cancer incidence	7	3311/44813	3252/44852	60	RR (random), 95% CI	1.01 (0.93 to 1.10)	0.78	⊕⊕⊕Ο Moderate
Total cancer mortality	7	932/41091	983/41116	0	RR (random), 95% CI	0.95 (0.87 to 1.04)	0.24	⊕⊕⊕⊕ High
All-cause mortality	8	2340/47361	2433/47392	0	RR (random), 95% CI	0.96 (0.91 to 1.02)	0.16	⊕⊕⊕⊕ High
**Major bleeding events**								
***Dose of aspirin***								
≤100 mg/d	16	1256/71279	876/71455	11	RR (fixed), 95% CI	1.44 (1.32 to 1.57)	<0.00001*	⊕⊕⊕⊕ High
100-300 mg/d	2	54/11441	34/11412	0	RR (fixed), 95% CI	1.58 (1.03 to 2.42)	0.04*	⊕⊕⊕⊕ High
>300 mg/d	3	76/15215	41/13116	0	RR (fixed), 95% CI	1.49 (1.02 to 2.18)	0.04*	⊕⊕⊕⊕ High
***Follow-up duration***								
1-5 years	12	801/58249	529/58016	0	RR (fixed), 95% CI	1.51 (1.35 to 1.69)	<0.00001*	⊕⊕⊕⊕ High
5-10 years	6	486/27602	358/25917	0	RR (fixed), 95% CI	1.34 (1.17 to 1.53)	<0.0001*	⊕⊕⊕⊕ High
>10 years	3	186/32230	136/32253	30	RR (fixed), 95% CI	1.37 (1.10 to 1.71)	0.005*	⊕⊕⊕⊕ High
**Total bleeding events**								
***Dose of aspirin***								
≤100 mg/d	11	850/43659	523/43744	40	RR (random), 95% CI	1.61 (1.37 to 1.89)	<0.00001*	⊕⊕⊕⊕ High
100-300 mg/d	4	3079/12719	2306/12693	40	RR (random), 95% CI	1.45 (1.13 to 1.85)	0.003*	⊕⊕⊕⊕ High
>300 mg/d	6	331/8765	123/5686	76	RR (random), 95% CI	1.72 (1.06 to 2.78)	0.03*	⊕⊕⊕Ο Moderate
***Follow-up duration***								
1-5 years	13	806/20952	406/19618	41	RR (fixed), 95% CI	1.77 (1.57 to 1.99)	<0.00001*	⊕⊕⊕⊕ High
5-10 years	4	3110/23919	2332/19658	26	RR (fixed), 95% CI	1.33 (1.26 to 1.39)	<0.00001*	⊕⊕⊕⊕ High
>10 years	2	207/21193	158/21219	0	RR (fixed), 95% CI	1.32 (1.07 to 1.62)	0.008*	⊕⊕⊕⊕ High

**Patient or population:** Participants without pre-existing cancer; **Setting:** Randomized clinical trials comparing aspirin versus no aspirin and reporting cancer outcomes, long-term aspirin use for the primary prevention of cancer? **Intervention:** Aspirin; **Comparison:** No aspirin. Abbreviations: **CVD**, cardiovascular diseases; **DM**, diabetes mellitus; **RCT**, randomized controlled trial; **RD**, risk difference; **RR**, risk ratio; **CI**, confidence interval; * Statistically significant.

## Abbreviations

CI: confidence interval
CVD: cardiovascular diseases
GRADE: The Grading of Recommendations Assessment Development and Evaluation Working Group Methodology
PRISMA: Preferred Reporting Items for Systematic Reviews and Meta-Analyses
RCT: randomized controlled trial
RD: risk difference
RR: risk ratio
TSA: Trial Sequential Analysis
WHO: World Health Organization
