# Supplementary material for: Long-term aspirin use for primary cancer prevention: An updated systematic review and subgroup meta-analysis of 29 randomized clinical trials
Source: J Cancer. 2020 Sep 14;11(21):6460–73. doi: 10.7150/jca.49001 (PMC7532493; doi:10.7150/jca.49001)
Supplement: Supplementary file 1 — Supplementary figures and tables. [file jcav11p6460s1.pdf]

Supplementary data

Supplementary Figure 1. PRISMA search diagram.

Supplementary Figure 2. Risk of bias graph.

Supplementary Figure 3. Risk of bias summary.

Supplementary Figure 4. Subgroup analysis based on aspirin dose showed that different daily doses of aspirin were not associated with significant reductions in total cancer incidence, total cancer mortality, or all-cause mortality. A) Total cancer incidence, B) total cancer mortality, C) all-cause mortality.

Supplementary Figure 5. The results of meta-regression analyses showing that the total cancer incidence, cancer mortality and all-cause mortality did not vary significantly with respect to the daily dose of aspirin (from ≤100 mg to >300 mg). A) Total cancer incidence, B) total cancer mortality, C) all-cause mortality.

Supplementary Figure 6. Subgroup analysis based on follow-up duration showed that different follow-up durations (1-5 years, 5-10 years, or > 10 years) were not associated with significant reductions in total cancer incidence, cancer mortality and all-cause mortality. A) Total cancer incidence, B) total cancer mortality, C) all-cause mortality.

Supplementary Figure 7. The meta-regression analysis showed that total cancer incidence, cancer mortality and all-cause mortality did not vary significantly with respect to follow-up duration (1-5 years to >10 years). A) Total cancer incidence, B) total cancer mortality, C) all-cause mortality.

Supplementary Figure 8. Subgroup analysis showing that the cancer incidence, cancer mortality and all-cause mortality were not reduced by low-dose aspirin (≤ 100 mg/day) use for more than five years.

Supplementary Figure 9. Subgroup analysis based on study populations showing that aspirin use did not decrease the total cancer incidence, total cancer mortality or all-cause mortality in five

different subgroups of participants, including the healthy population, patients with diabetes mellitus, participants with CVD or an increased risk of CVD, individuals with an increased risk of cancer, or patients with peripheral arterial disease or venous thromboembolism. A) Total cancer incidence, B) total cancer mortality, C) all-cause mortality.

Supplementary Figure 10. Subgroup analysis based on the daily dose of aspirin showed that all three different daily doses of aspirin (≤ 100 mg, 100-300 mg, or > 300 mg daily) significantly increased the risk of major bleeding and total bleeding events. A) Major bleeding, B) total bleeding events.

Supplementary Figure 11. Subgroup analysis based on follow-up duration showed that the risk of major bleeding and total bleeding events significantly increased with different follow-up durations (1-5 years, 5-10 years, or > 10 years). A) Major bleeding, B) total bleeding events.

Supplementary Figure 12. Trial sequential analysis indicated that aspirin use was not significantly superior to no aspirin, and the cumulated sample size of all the RCTs reached the required information size (IS) needed for a conclusive and reliable meta-analysis, suggesting that the findings of the meta-analysis were robust for the cancer incidence.

Supplementary Figure 13. The funnel plots of total cancer incidence (the primary outcome).

Supplementary Table 1. The methodologic quality of the included trials was assessed using the Cochrane risk-of-bias tool.

Supplementary Table 2. GRADE evidence profile: long-term aspirin use for cancer primary prevention.

Supplementary Table 3. Sensitivity analyses.

Supplementary Figure 1: PRISMA diagram of searching

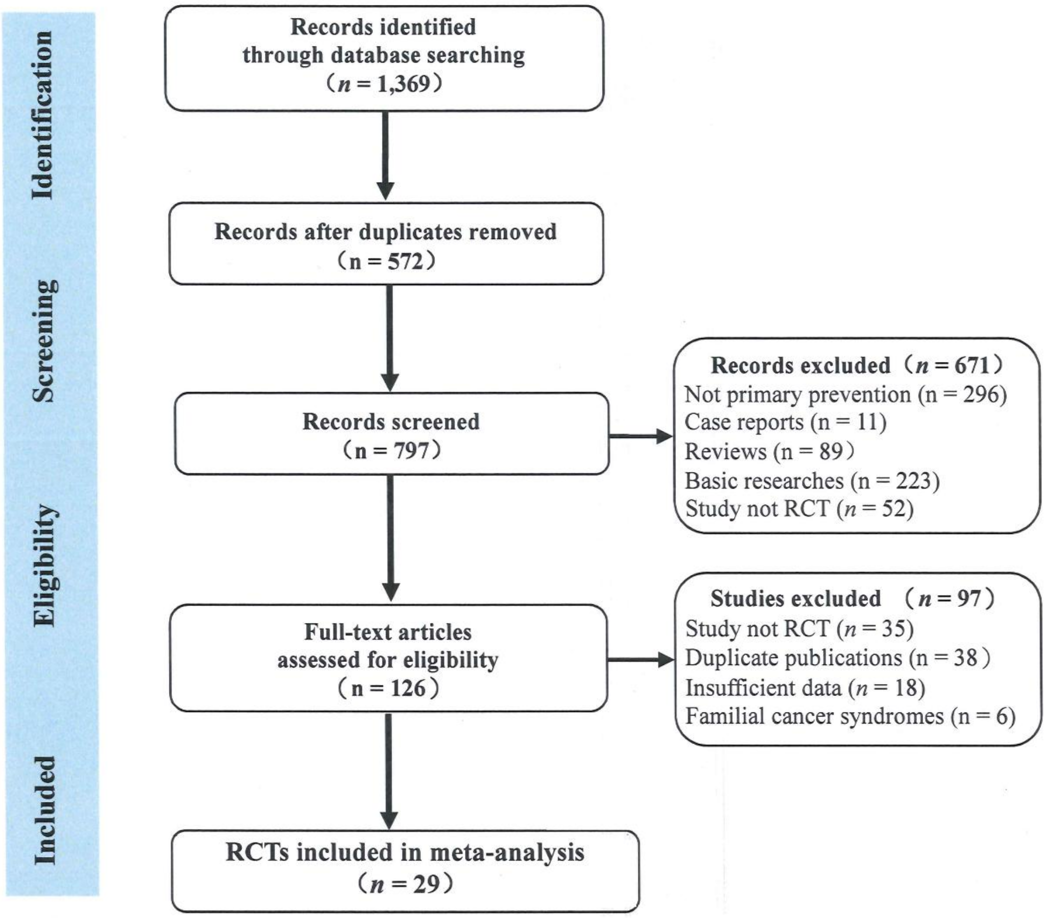

RCT, randomised controlled trial;

PRISMA, Preferred Reporting Items for Systematic Reviews and Meta-Analyses.

Supplementary Figure 2: Risk of bias graph.

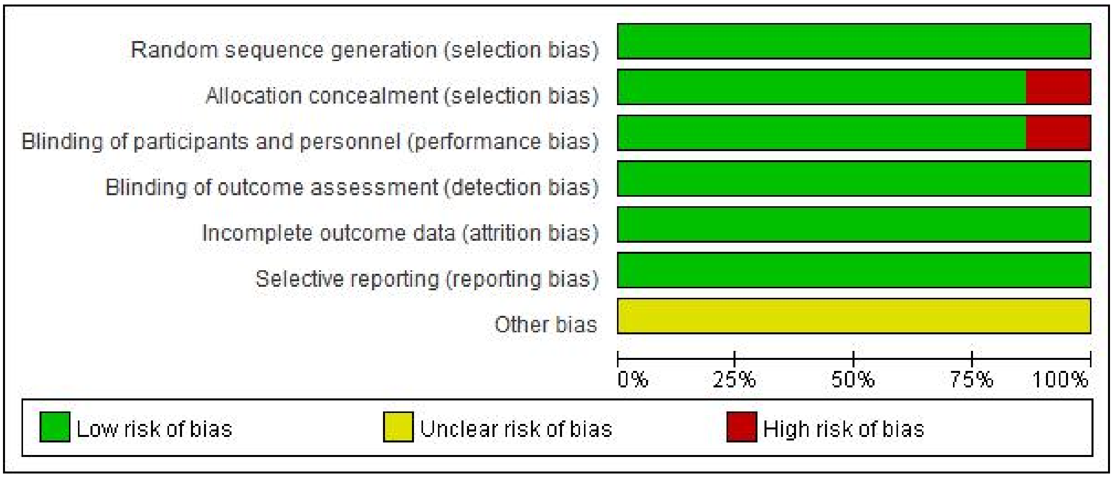

Supplementary Figure 3: Risk of bias summary.

	Random sequence generation (selection bias)	Allocation concealment (selection bias)	Blinding of participants and personnel (performance bias)	Blinding of outcome assessment (detection bias)	Incomplete outcome data (attrition bias)	Selective reporting (reporting bias)	Other bias
AAA, 2010	+	+	+	+	+	+	?
AFPPS, 2003	+	+	+	+	+	+	?
AMIS, 1980	+	+	+	+	+	+	?
ARRIVE, 2018	+	+	+	+	+	+	?
ASCEND, 2018	+	+	+	+	+	+	?
ASPIRE, 2012	+	+	+	+	+	+	?
ASPREE, 2018	+	+	+	+	+	+	?
BDS, 1988	+	-	-	+	+	+	?
CDPA, 1976	+	+	+	+	+	+	?
CLIPS, 2007	+	+	+	+	+	+	?
DAMAD, 1989	+	+	+	+	+	+	?
EAFT, 1993	+	+	+	+	+	+	?
ESPS-2, 1996	+	+	+	+	+	+	?
ETDRS, 1992	+	+	+	+	+	+	?
HOT, 1998	+	+	+	+	+	+	?
JPAD, 2008/2017/2018	+	-	-	+	+	+	?
JPPP, 2017	+	-	-	+	+	+	?
PARIS, 1980	+	+	+	+	+	+	?
PHS, 1989/1998	+	+	+	+	+	+	?
POPADAD, 2008	+	+	+	+	+	+	?
PPP, 2001	+	-	-	+	+	+	?
REDUCE, 2015	+	+	+	+	+	+	?
SALT, 1991	+	+	+	+	+	+	?
SAPAT, 1992	+	+	+	+	+	+	?
seAFOod, 2018	+	+	+	+	+	+	?
TPT, 1998	+	+	+	+	+	+	?
ukCAP, 2008	+	+	+	+	+	+	?
UK-TIA, 1991	+	+	+	+	+	+	?
WHS, 2005	+	+	+	+	+	+	?

Supplementary Figure 4. Subgroup analysis based on aspirin dose showed that different daily doses of aspirin were not associated with significant reductions in total cancer incidence, total cancer mortality, or all-cause mortality. A) Total cancer incidence, B) total cancer mortality, C) all-cause mortality.

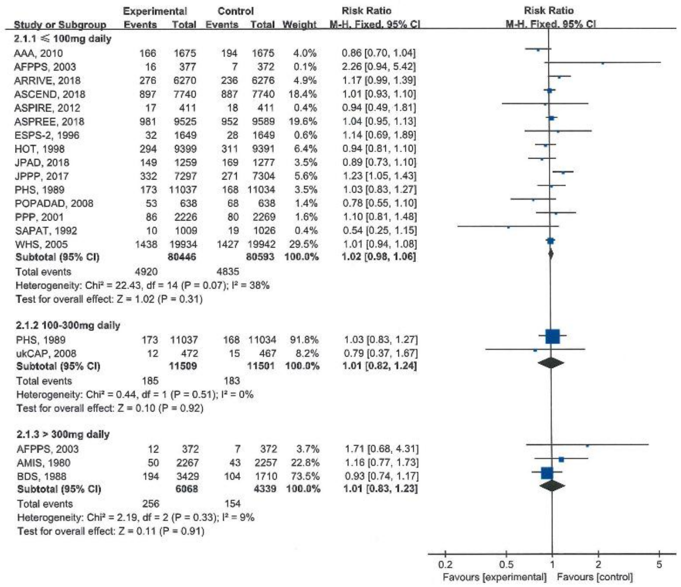

A

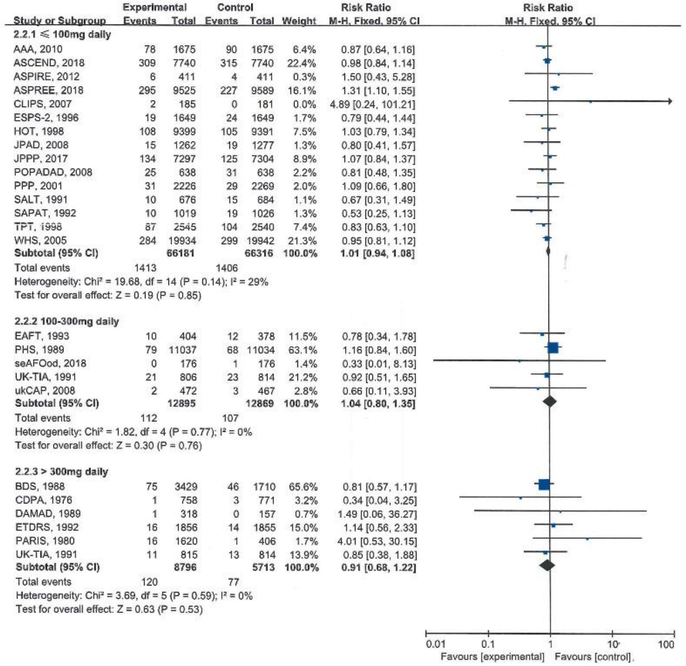

B

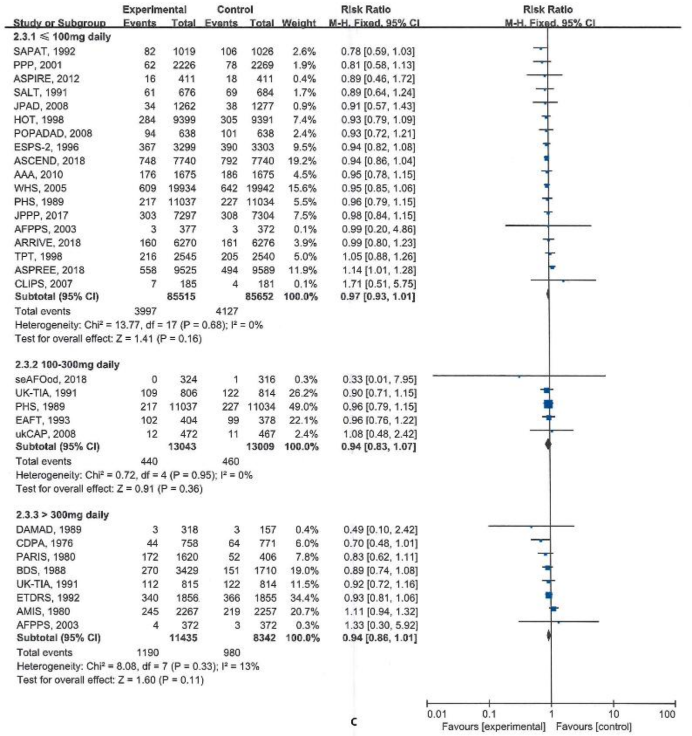

C

Supplementary Figure 5. The results of meta-regression analyses showing that the total cancer incidence, cancer mortality and all-cause mortality did not vary significantly with respect to the daily dose of aspirin (from ≤100 mg to >300 mg). A) Total cancer incidence, B) total cancer mortality, C) all-cause mortality.

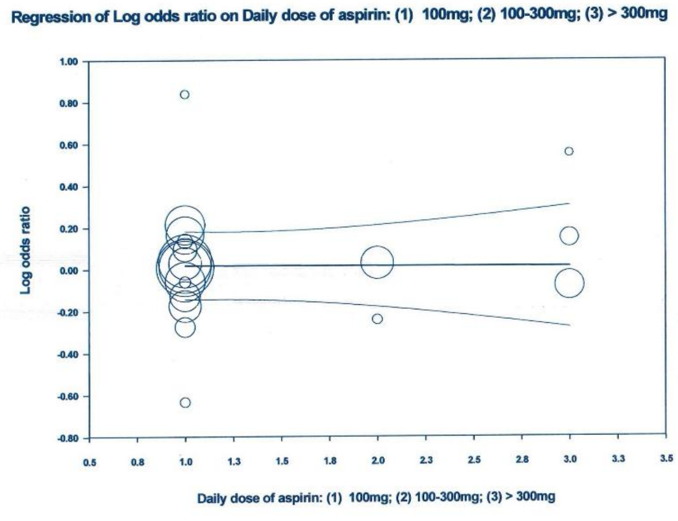

A

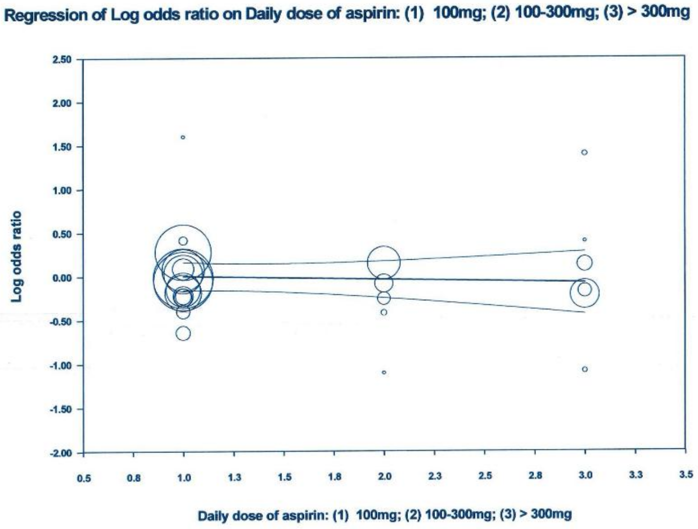

B

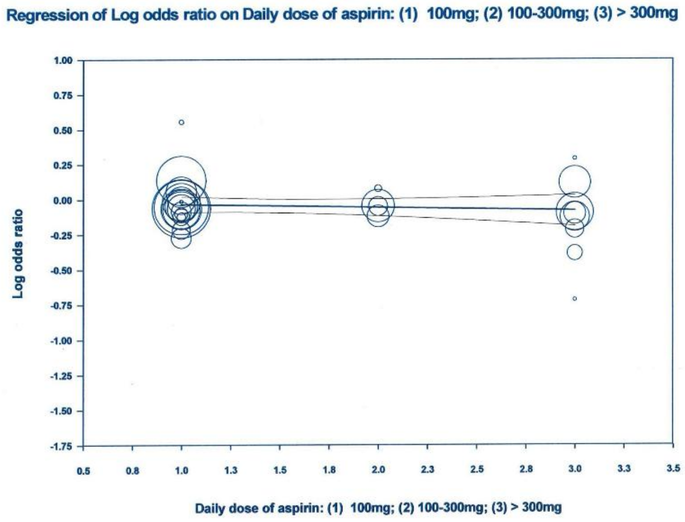

C

Supplementary Figure 6. Subgroup analysis based on follow-up duration showed that different follow-up durations (1-5 years, 5-10 years, or > 10 years) were not associated with significant reductions in total cancer incidence, cancer mortality and all-cause mortality. A) Total cancer incidence, B) total cancer mortality, C) all-cause mortality.

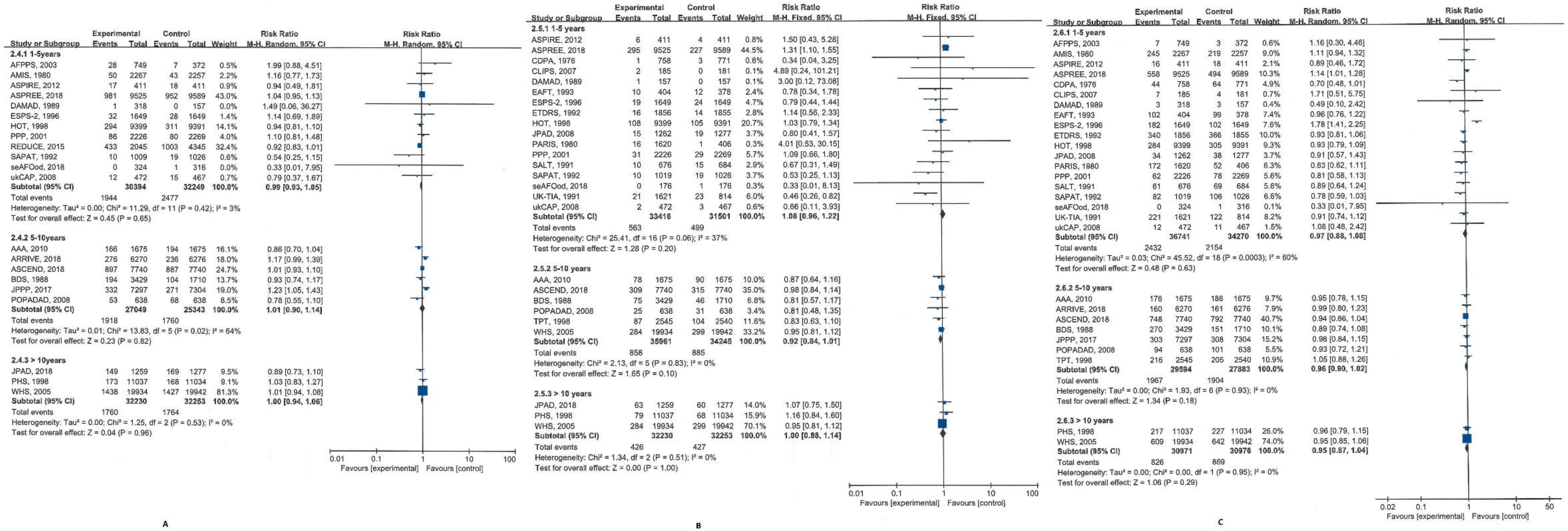

Supplementary Figure 7. The meta-regression analysis showed that total cancer incidence, cancer mortality and all-cause mortality did not vary significantly with respect to follow-up duration (1-5 years to >10 years). A) Total cancer incidence, B) total cancer mortality, C) all-cause mortality.

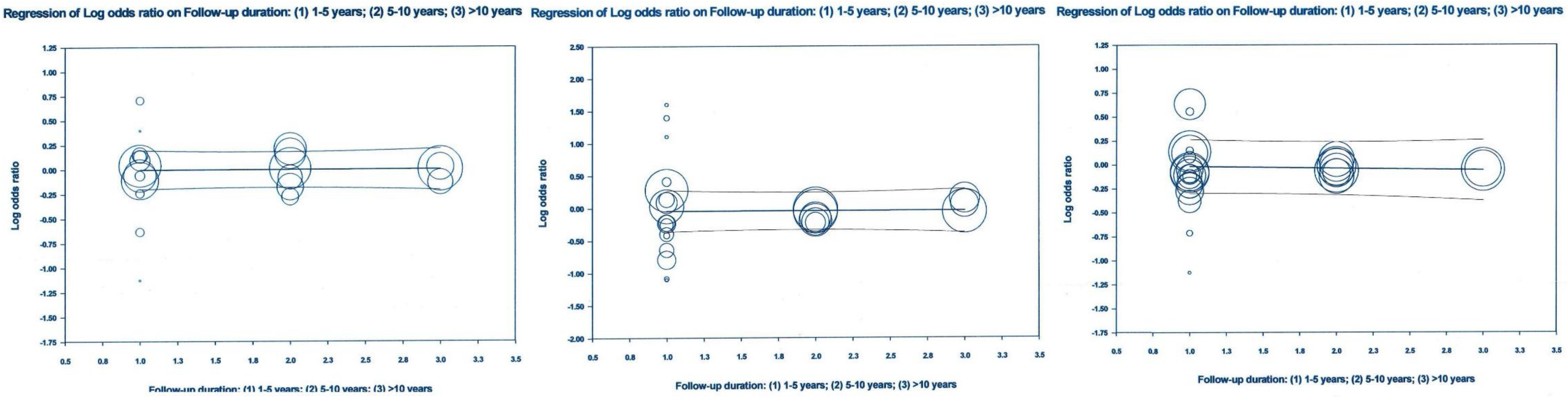

A B C

Supplementary Figure 8. Subgroup analysis showing that the cancer incidence, cancer mortality and all-cause mortality were not reduced by low-dose aspirin (≤ 100 mg/day) use for more than five years.

Study or Subgroup	Experimental Events	Experimental Total	Control Events	Control Total	Weight	Risk Ratio M-H, Random, 95% CI
**2.7.1 total cancer Incidence**						
AAA, 2010	166	1675	194	1675	11.4%	0.86 [0.70, 1.04]
ARRIVE, 2018	276	6270	236	6276	13.3%	1.17 [0.99, 1.39]
ASCEND, 2018	897	7740	887	7740	21.6%	1.01 [0.93, 1.10]
JPAD, 2008	149	1259	169	1277	10.7%	0.89 [0.73, 1.10]
JPPP, 2017	332	7297	271	7304	14.4%	1.23 [1.05, 1.43]
POPADAD, 2008	53	638	68	638	5.2%	0.78 [0.55, 1.10]
WHS, 2005	1438	19934	1427	19942	23.3%	1.01 [0.94, 1.08]
**Subtotal (95% CI)**		**44813**		**44852**	**100.0%**	**1.01 [0.93, 1.10]**
Total events	3311		3252			
Heterogeneity: Tau² = 0.01; Chi² = 14.96, df = 6 (P = 0.02); I² = 60%						
Test for overall effect: Z = 0.27 (P = 0.78)						
**2.7.2 total cancer mortality**						
AAA, 2010	78	1675	90	1675	8.9%	0.87 [0.64, 1.16]
ASCEND, 2018	309	7740	315	7740	33.0%	0.98 [0.84, 1.14]
JPAD, 2008	15	1262	19	1277	1.7%	0.80 [0.41, 1.57]
JPPP, 2017	134	7297	125	7304	13.4%	1.07 [0.84, 1.37]
POPADAD, 2008	25	638	31	638	2.9%	0.81 [0.48, 1.35]
TPT, 1998	87	2545	104	2540	10.0%	0.83 [0.63, 1.10]
WHS, 2005	284	19934	299	19942	30.0%	0.95 [0.81, 1.12]
**Subtotal (95% CI)**		**41091**		**41116**	**100.0%**	**0.95 [0.87, 1.04]**
Total events	932		983			
Heterogeneity: Tau² = 0.00; Chi² = 2.98, df = 6 (P = 0.81); I² = 0%						
Test for overall effect: Z = 1.18 (P = 0.24)						
**2.7.3 all-cause mortality**						
AAA, 2010	176	1675	186	1675	7.9%	0.95 [0.78, 1.15]
ARRIVE, 2018	160	6270	161	6276	6.4%	0.99 [0.80, 1.23]
ASCEND, 2018	748	7740	792	7740	33.3%	0.94 [0.86, 1.04]
JPAD, 2008	34	1262	38	1277	1.4%	0.91 [0.57, 1.43]
JPPP, 2017	303	7297	308	7304	12.4%	0.98 [0.84, 1.15]
POPADAD, 2008	94	638	101	638	4.5%	0.93 [0.72, 1.21]
TPT, 1998	216	2545	205	2540	8.9%	1.05 [0.88, 1.26]
WHS, 2005	609	19934	642	19942	25.1%	0.95 [0.85, 1.06]
**Subtotal (95% CI)**		**47361**		**47392**	**100.0%**	**0.96 [0.91, 1.02]**
Total events	2340		2433			
Heterogeneity: Tau² = 0.00; Chi² = 1.45, df = 7 (P = 0.98); I² = 0%						
Test for overall effect: Z = 1.40 (P = 0.16)						

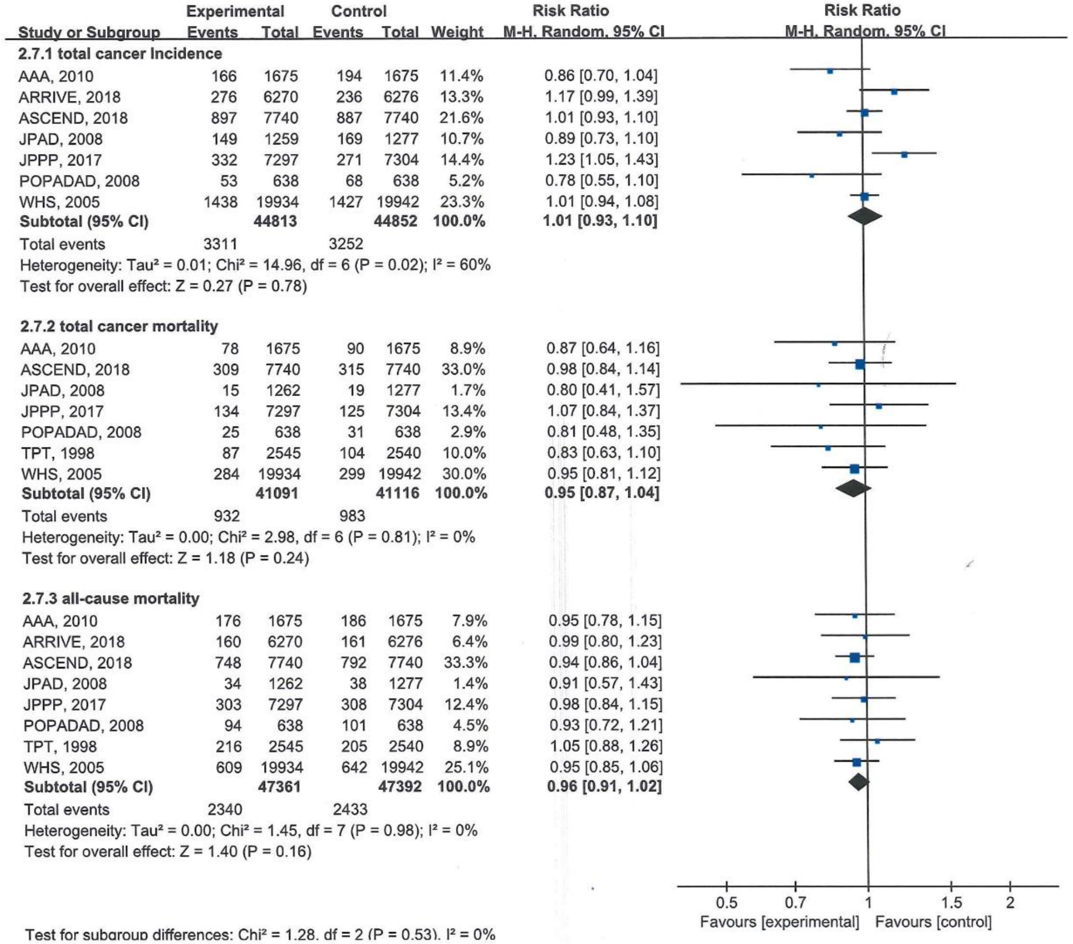

Test for subgroup differences: Chi² = 1.28, df = 2 (P = 0.53), I² = 0%

Supplementary Figure 9. Subgroup analysis based on study populations showing that aspirin use did not decrease the total cancer incidence, total cancer mortality or all-cause mortality in five different subgroups of participants, including the healthy population, patients with diabetes mellitus, participants with CVD or an increased risk of CVD, individuals with an increased risk of cancer, or patients with peripheral arterial disease or venous thromboembolism. A) Total cancer incidence, B) total cancer mortality, C) all-cause mortality.

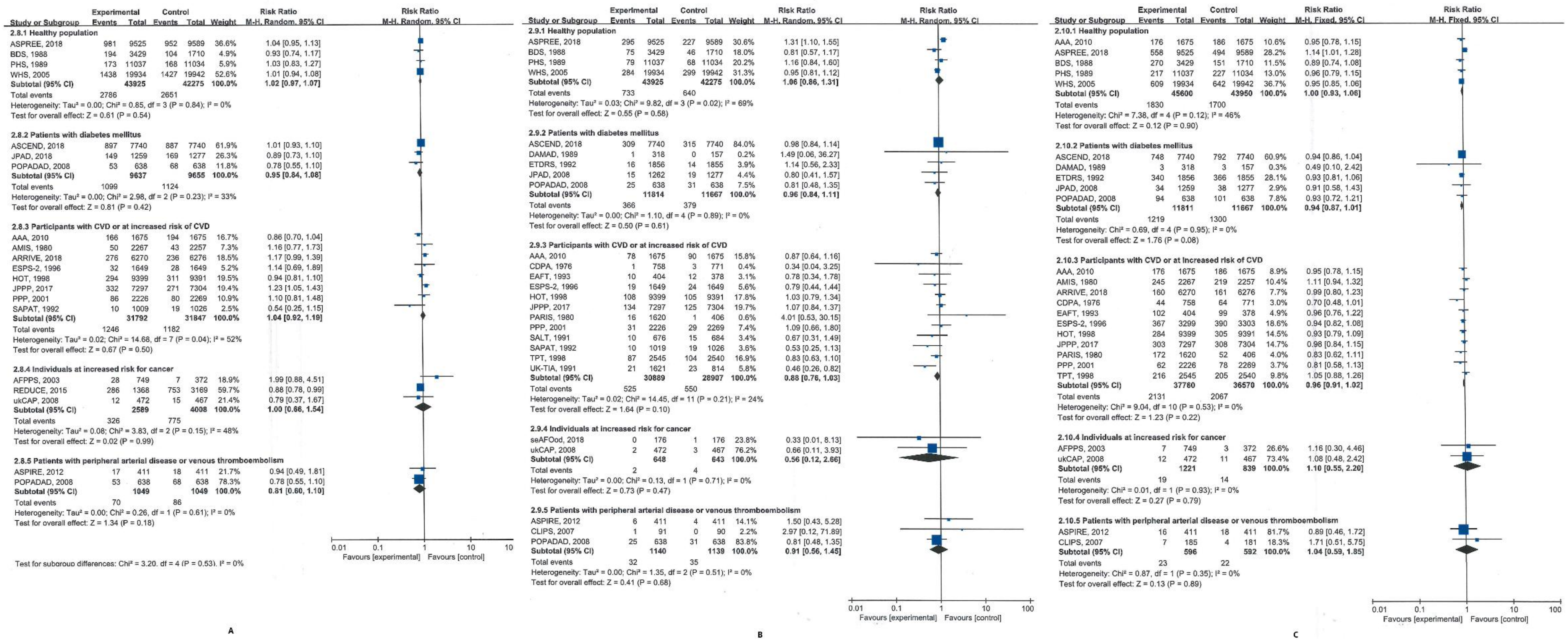

Supplementary Figure 10. Subgroup analysis based on the daily dose of aspirin showed that all three different daily doses of aspirin (≤ 100 mg, 100-300 mg, or > 300 mg daily) significantly increased the risk of major bleeding and total bleeding events. A) Major bleeding, B) total bleeding events.

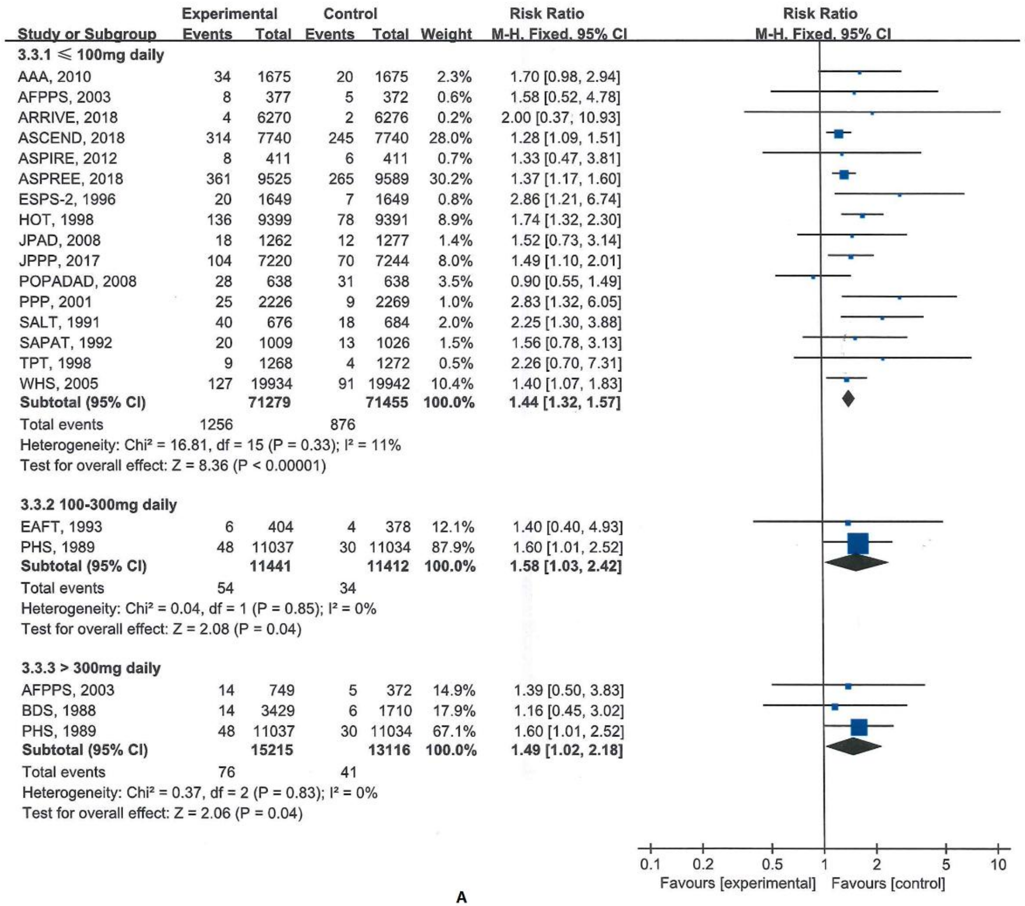

A

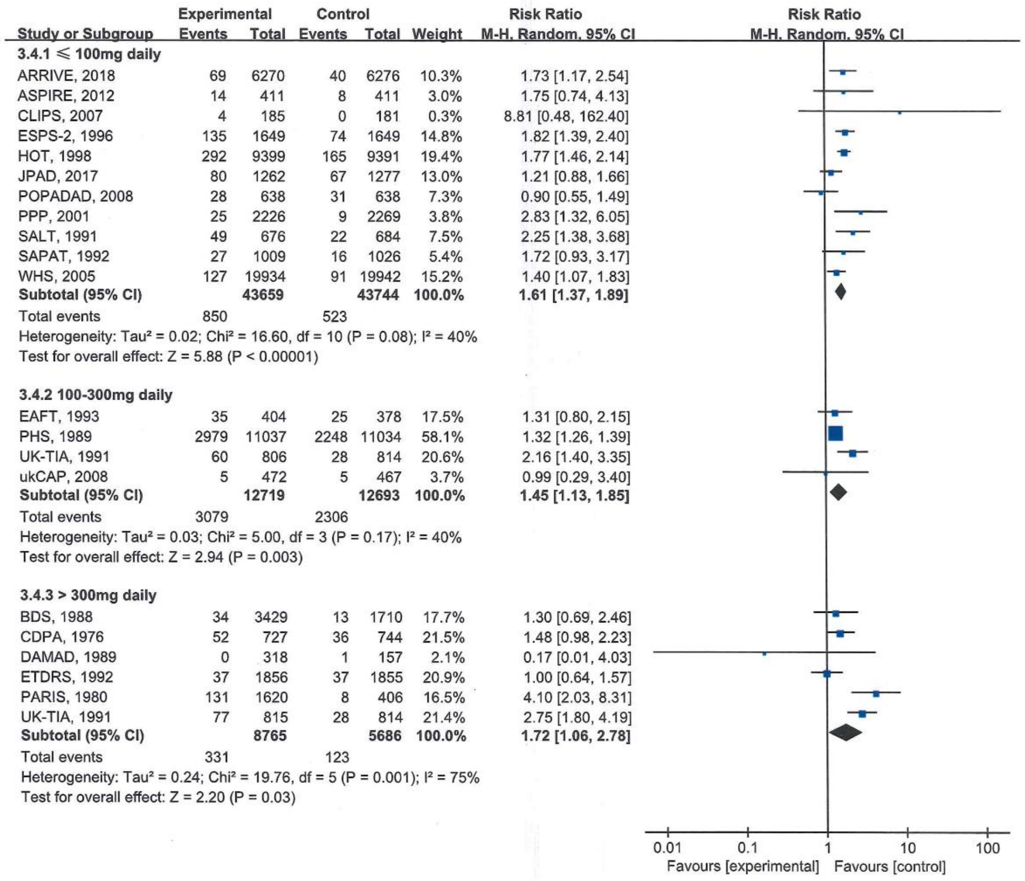

B

Supplementary Figure 11. Subgroup analysis based on follow-up duration showed that the risk of major bleeding and total bleeding events significantly increased with different follow-up durations (1-5 years, 5-10 years, or > 10 years). A) Major bleeding, B) total bleeding events.

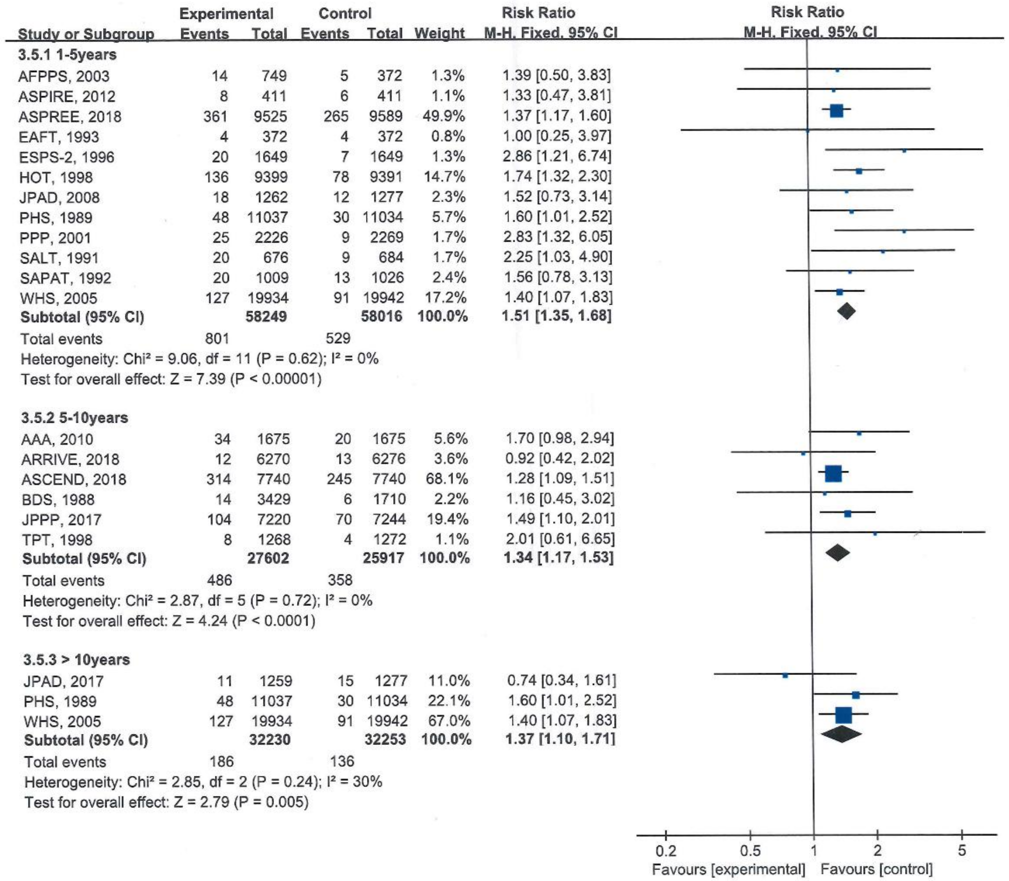

A

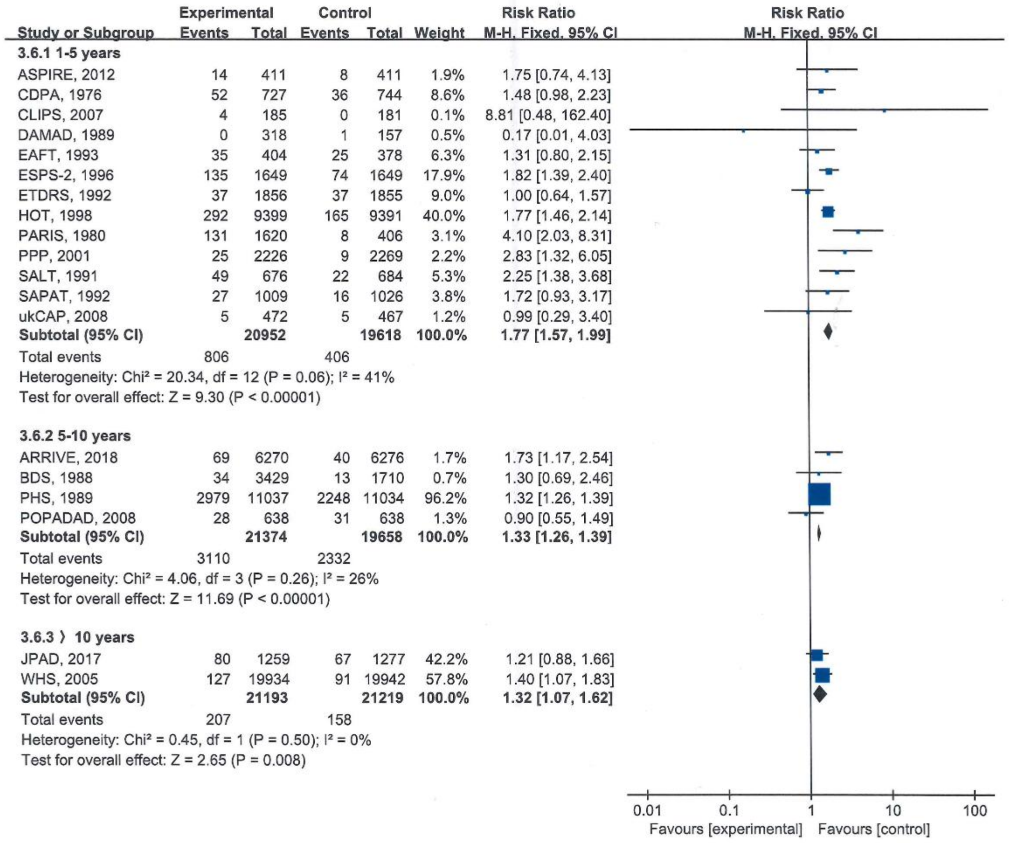

B

Supplementary Figure 12. Trial sequential analysis indicated that aspirin use was not significantly superior to no aspirin, and the cumulated sample size of all the RCTs reached the required information size (IS) needed for a conclusive and reliable meta-analysis, suggesting that the findings of the meta-analysis were robust for the cancer incidence.

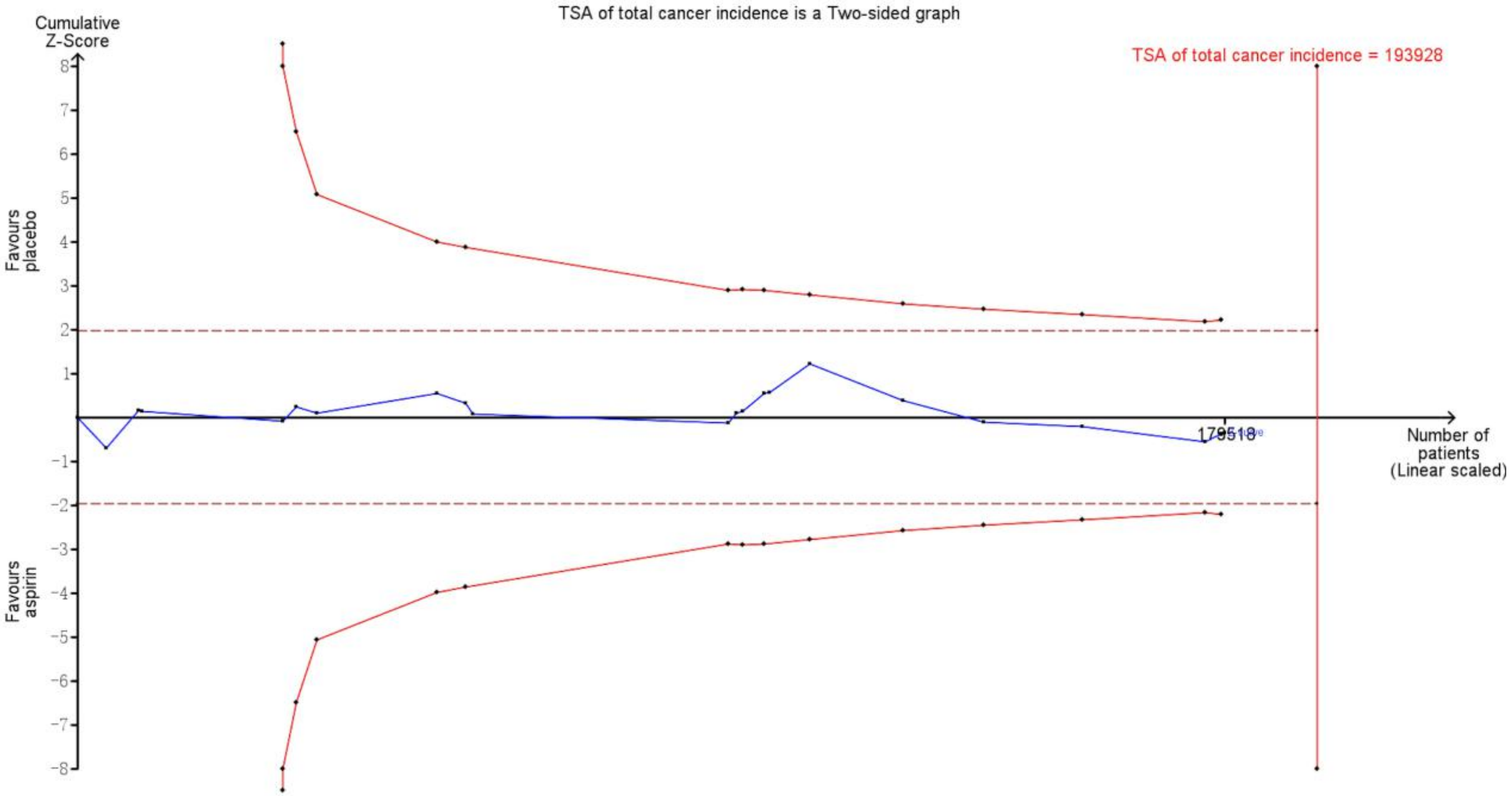

Supplementary Figure 13. The funnel plots of total cancer incidence (the primary outcome).

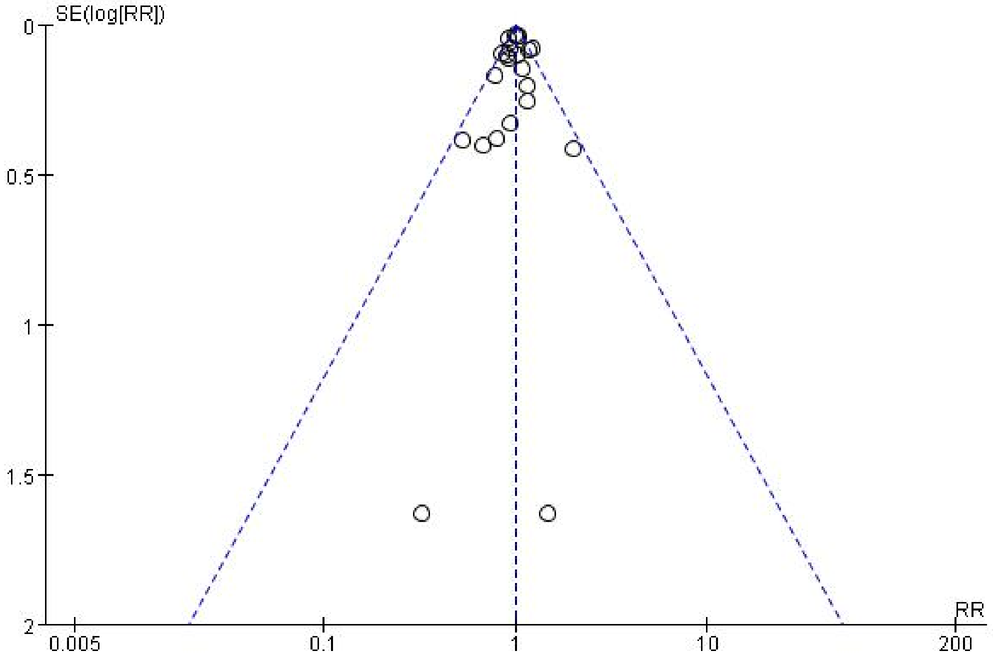

Supplementary Table 1: The methodologic quality of the included trials assessed using the Cochrane risk of bias tool.

Reference	Random sequence generation	Allocation concealment	Blinding of participants and personnel	Blinding of outcome assessment	Incomplete outcome data	Selective reporting	Other bias
AAA, 2010 [1]	+	+	+	+	+	+	?
AFPPS, 2003 [2]	+	+	+	+	+	+	?
AMIS, 1980 [3]	+	+	+	+	+	+	?
ARRIVE, 2018 [4]	+	+	+	+	+	+	?
ASCEND, 2018 [5]	+	+	+	+	+	+	?
ASPIRE, 2012 [6]	+	+	+	+	+	+	?
ASPREE, 2018 [7]	+	+	+	+	+	+	?
BDS,1988 [8]	+	-	-	+	+	+	?
CDPA, 1980 [9]	+	+	+	+	+	+	?
CLIPS, 2007 [10]	+	+	+	+	+	+	?
DAMAD, 1989 [11]	+	+	+	+	+	+	?
EAFT, 1993 [12]	+	+	+	+	+	+	?
ESPS-2, 1996 [13]	+	+	+	+	+	+	?
ETDRS, 1992 [14]	+	+	+	+	+	+	?
HOT, 1998 [15]	+	+	+	+	+	+	?
JPAD, 2008 [16]/ 2017[17]/2018 [18]	+	-	-	+	+	+	?
JPPP, 2014 [19]	+	-	-	+	+	+	?
PARIS, 1980 [20]	+	+	+	+	+	+	?
PHS, 1989 [21]/1998 [22]	+	+	+	+	+	+	?
POPADAD, 2008 [23]	+	+	+	+	+	+	?
PPP, 2001 [24]	+	-	-	+	+	+	?
REDUCE, 2015 [25]	+	+	+	+	+	+	?
SALT, 1991 [26]	+	+	+	+	+	+	?
SAPAT, 1992 [27]	+	+	+	+	+	+	?
seAFOod, 2018 [28]	+	+	+	+	+	+	?
TPT, 1998 [29]	+	+	+	+	+	+	?
ukCAP, 2008 [30]	+	+	+	+	+	+	?
UK-TIA, 1991 [31]	+	+	+	+	+	+	?
WHS, 2005 [32]	+	+	+	+	+	+	?

+ = low risk of bias; ? = unclear risk of bias; - = high risk of bias.

AAA, Aspirin for Asymptomatic AtherosclerosisTrial; **AFPPS**, The Aspirin/Folate Polyp Prevention Study; **AMIS**, Aspirin Myocardial Infarction Study; **ARRIVE**, Aspirin to Reduce Risk of Initial Vascular Events; **ASCEND**, A Study of Cardiovascular Events in Diabetes; **ASPIRE**, Aspirin to Prevent Recurrent Venous Thromboembolism trial; **ASPREE**, Aspirin in Reducing Events in the Elderly; **BDS**, British Doctors Study; **CDPA**, Coronary Drug Project Research; **CLIPS**, Critical Leg Ischaemia Prevention Study; **DAMAD**, the Dipyridamole Aspirin Microangiopathy of Diabetes study; **EAFT**, European atrial fibrillation trial; **ESPS-2**, European Stroke Prevention Study 2; **ETDRS**, Early Treatment Diabetic Retinopathy Study; HOT, Hypertension Optimal Treatment; **JPAD**, Japanese Primary Prevention of Atherosclerosis with Aspirin for Diabetes; **JPPP**, Japanese Primary Prevention Project; **PARIS**, The Persantine-Aspirin Reinfarction Study; **PHS**, Physicians'Health Study; **POPADAD**, Prevention of Arterial Disease and Diabetes; **PPP**, Primary Prevention Project; **REDUCE**, the Reduction by Dutasteride of Prostate Cancer Events study; **SAPAT**, The Swedish Angina Pectoris Aspirin Trial; **SALT**, Swedish Aspirin Low-dose Trial; **seAFOod**, The Systematic Evaluation of Aspirin and Fish Oil Polyp Prevention Trial; **TPT**, Thrombosis Prevention Trial; **ukCAP**, The United Kingdom Colorectal Adenoma Prevention; **UK-TIA**, The United Kingdom transient ischaemic attack; **WHS**, Women's Health Study.

Supplementary Table 2: GRADE evidence profile：long-term aspirin use for cancer primary prevention

Indicators (Trials)			Quality assessment					Summary of findings				
								No. of patients		Relative risk (95% CI)	Absolute effects	Quality
			Limitations	Inconsistency	Indirectness	Imprecision	Publication bias	Aspirin	No apsirin		Risk difference (95% CI)	
Total cancer incidence (21)			No serious [a]	No serious	No serious	No serious	Undetected	5622/89673 (6.27%)	6001/89845 (6.68%)	1.01 (0.97 to 1.04)[#]	4 fewer per 1000 (-6 to -2)	⊕⊕⊕⊕ High
Total cancer mortality (25)			No serious [a]	No serious	No serious	No serious	Undetected	1634/88020 (1.86%)	1577/84224 (1.87%)	1.00 (0.93 to 1.07))[#]	0 more per 1000 (-1 to 1)	⊕⊕⊕⊕ High
All-cause mortality (28)			No serious [a]	No serious	No serious	No serious	Undetected	5225/97303 (5.37%)	4927/93129 (5.29%)	0.98 (0.94 to 1.02))[#]	1 more per 1000 (-1 to 3)	⊕⊕⊕⊕ High
Major bleeding events (18)			No serious [a]	No serious	No serious	No serious	Undetected	1288/85851 (1.49%)	887/83933 (1.05%)	1.44 (1.32 to 1.57))[#]	4 more per 1000 (3 to 5)	⊕⊕⊕⊕ High
Total bleeding events (19)			No serious [b]	Serious [e]	No serious	No serious	Undetected	4123/63519 (6.49%)	2896/60495 (4.79%)	1.52 (1.33 to 1.74)[*]	17 more per 1000 (15 to 20)	⊕⊕⊕O Moderate
**Subgroup analyses**												
Total cancer incidence	Dose of aspirin	≤ 100mg daily (15)	No serious	No serious	No serious	No serious	Undetected	4920/80446 (6.12%)	4835/80593 (6.00%)	1.02 (0.98 to 1.06)[#]	1 more per 1000 (-1 to 4)	⊕⊕⊕⊕ High
		100-300mg daily (2)	No serious	No serious	No serious	No serious	Undetected	185/11509 (1.61%)	183/11501 (1.59%)	1.01 (0.82 to 1.24)[#]	0 more per 1000 (-3 to 3)	⊕⊕⊕⊕ High
		> 300mg daily (3)	No serious	No serious	No serious	No serious	Undetected	256/6068 (4.22%)	154/4349 (3.54%)	1.01 (0.83 to 1.23)[#]	7 more per 1000 (-1 to 14)	⊕⊕⊕⊕ High
	Follow-up duration	1-5 years (12)	No serious	No serious	No serious	No serious	Undetected	1944/30394 (6.40%)	2477/32249 (7.68%)	0.99 (0.93 to 1.05)[*]	13 fewer per 1000 (-17 to -9)	⊕⊕⊕⊕ High
		5-10 years (6)	No serious	Serious [e]	No serious	No serious	Undetected	1918/27049 (7.09%)	1706/25343 (6.73%)	1.01 (0.90 to 1.14)[*]	2 more per 1000 (-3 to 6)	⊕⊕⊕O Moderate
		>10 years (3)	No serious	No serious	No serious	No serious	Undetected	1760/32230 (5.46%)	1764/32253 (5.47%)	1.00 (0.94 to 1.06)[*]	0 fewer per 1000 (-4 to 3)	⊕⊕⊕⊕ High
	Study populations	Healthy population (4)	No serious	No serious	No serious	No serious	Undetected	2786/43925 (6.34%)	2651/42275 (6.27%)	1.02 (0.97 to 1.07)[*]	1 more per 1000 (-3 to 4)	⊕⊕⊕⊕ High
		With DM (3)	No serious	No serious	No serious	No serious	Undetected	1099/9637 (11.40%)	1124/9655 (11.64%)	0.95 (0.84 to 1.08)[*]	2 fewer per 1000 (-11 to 7)	⊕⊕⊕⊕ High
		With CVD or at increased risk of CVD (8)	No serious	Serious [e]	No serious	No serious	Undetected	1246/31972 (3.92%)	1182/31847 (3.71%)	1.04 (0.92 to 1.19)[*]	2 more per 1000 (-1 to 5)	⊕⊕⊕O Moderate
		At increased risk of cancer (3)	No serious	No serious	No serious	No serious	Undetected	326/2589 (12.59%)	775/4008 (19.34%)	1.00 (0.66 to 1.54)[*]	67 fewer per 1000 (-85 to -50)	⊕⊕⊕⊕ High
		With peripheral arterial disease or venous thromboembolism (2)	No serious	No serious	No serious	No serious	Undetected	70/1049 (6.67%)	86/1049 (8.20%)	0.81 (0.60 to 1.10)[*]	15 fewer per 1000 (-38 to 7)	⊕⊕⊕⊕ High
Total cancer mortality	Dose of aspirin	≤ 100mg daily (15)	No serious	No serious	No serious	No serious	Undetected	1413/66181 (2.14%)	1406/66316 (2.12%)	1.01 (0.94 to 1.08)[#]	0 more per 1000 (-1 to 2)	⊕⊕⊕⊕ High
		100-300mg daily (5)	No serious	No serious	No serious	No serious	Undetected	112/12895 (0.87%)	107/12869 (0.83%)	1.04(0.80 to 1.35)[#]	0 more per 1000 (-2 to 3)	⊕⊕⊕⊕ High
		> 300mg daily(6)	No serious	No serious	No serious	No serious	Undetected	120/8796 (1.36%)	77/5713 (1.35%)	0.91 (0.68 to 1.22)[#]	0 more per 1000 (-4 to 4)	⊕⊕⊕⊕ High
	Follow-up duration	1-5 years (17)	No serious	No serious	No serious	No serious	Undetected	563/33416 (1.68%)	499/31501 (1.58%)	1.08 (0.96 to 1.22)[#]	1 more per 1000 (-1 to 3)	⊕⊕⊕⊕ High
		5-10 years (6)	No serious	No serious	No serious	No serious	Undetected	858/35961 (1.05%)	885/34245 (2.58%)	0.92 (0.84 to 1.01)[#]	2 fewer per 1000 (-4 to 0)	⊕⊕⊕⊕ High

		>10 years (3)	No serious	No serious	No serious	No serious	Undetected	426/32230 (1.17%)	427/32253 (1.32%)	1.00 (0.88 to 1.14)#	0 fewer per 1000 (-2 to 2)	⊕⊕⊕⊕ High
	Study populations	Healthy population (4)	No serious	Serious [e]	No serious	No serious	Undetected	733/43925 (1.67%)	640/42275 (1.51%)	1. 06 (0.86 to 1.31)*	2 more per 1000 (0 to 3)	⊕⊕⊕O Moderate
		With DM (5)	No serious	No serious	No serious	No serious	Undetected	366/11814 (3.10%)	379/11667 (3.25%)	0.96 (0.84 to 1.11)*	1 fewer per 1000 (-6 to 4)	⊕⊕⊕⊕ High
		With CVD or at increased risk of CVD (12)	No serious	No serious	No serious	No serious	Undetected	525/30889 (1.70%)	550/28907 (1.90%)	0.88 (0.76 to 1.03)*	2 fewer per 1000 (-4 to 1)	⊕⊕⊕⊕ High
		At increased risk of cancer (2)	No serious	No serious	No serious	No serious	Undetected	2/648 (0.31%)	4/643 (0.62%)	0.56 (0.12 to 2.66)*	3 fewer per 1000 (-11 to 4)	⊕⊕⊕⊕ High
		With peripheral arterial disease or venous thromboembolism (3)	No serious	No serious	No serious	No serious	Undetected	32/1140 (2.81%)	35/1139 (3.07%)	0.91 (0.56 to 1.45)*	3 fewer per 1000 (-17 to 11)	⊕⊕⊕⊕ High
All-cause mortality	Dose of aspirin	≤ 100mg daily (18)	No serious	No serious	No serious	No serious	Undetected	3997/85512 (4.67%)	4127/85652 (4.82%)	0.97 (0.93 to 1.01)#	1 fewer per 1000 (-4 to 1)	⊕⊕⊕⊕ High
		100-300mg daily (5)	No serious	No serious	No serious	No serious	Undetected	440/13043 (3.37%)	460/13009 (3.54%)	0.94 (0.83 to 1.07)#	2 fewer per 1000 (-6 to 3)	⊕⊕⊕⊕ High
		> 300mg daily (8)	No serious	No serious	No serious	No serious	Undetected	1190/11435 (10.41%)	980/8342 (11.75%)	0.94 (0.86 to 1.01)#	13 less per 1000 (-22 to -5)	⊕⊕⊕⊕ High
	Follow-up duration	1-5 years (19)	No serious	Serious [e]	No serious	No serious	Undetected	2432/36741 (6.76%)	2154/34270 (6.59%)	0.97 (0.88 to 1.08)*	4 more per 1000 (0 to 7)	⊕⊕⊕O Moderate
		5-10 years (7)	No serious	No serious	No serious	No serious	Undetected	1967/29549 (6.65%)	1904/27883 (6.41%)	0.96 (0.90 to 1.02)*	2 fewer per 1000 (-6 to 2)	⊕⊕⊕⊕ High
		>10 years (2)	No serious	No serious	No serious	No serious	Undetected	826/30971 (2.67%)	869/30976 (2.81%)	0.95 (0.87 to 1.04)*	1 fewer per 1000 (-4 to 1)	⊕⊕⊕⊕ High
	Study populations	Healthy population (5)	No serious	No serious	No serious	No serious	Undetected	1830/45600 (4.00%)	1700/43950 (3.90%)	1.00 (0.93 to 1.06)#	2 more per 1000 (-1 to 4)	⊕⊕⊕⊕ High
		With DM (5)	No serious	No serious	No serious	No serious	Undetected	1219/11811 (10.32%)	1300/11667 (11.14%)	0.94 (0.87 to 1.01)#	8 fewer per 1000 (-16 to 0)	⊕⊕⊕⊕ High
		With CVD or at increased risk of CVD (11)	No serious	No serious	No serious	No serious	Undetected	2131/37760 (5.64%)	2067/36570 (5.65%)	0.96(0.91 to 1.02)#	0 fewer per 1000 (-3 to 3)	⊕⊕⊕⊕ High
		At increased risk of cancer (2)	No serious	No serious	No serious	No serious	Undetected	19/1221 (1.56%)	14/839 (1.67%)	1.10 (0.55 to 2.20)#	1 fewer per 1000 (-12 to 10)	⊕⊕⊕⊕ High
		With peripheral arterial disease or venous thromboembolism (2)	No serious	No serious	No serious	No serious	Undetected	23/596 (3.86%)	22/592 (3.72%)	1.04 (0.59 to 1.85)#	1 more per 1000 (-20 to 23)	⊕⊕⊕⊕ High
Total cancer incidence (7)	Dose of aspirin and Follow-up duration	Aspirin ≤ 100mg/d for more than five years	No serious	Serious [e]	No serious	No serious	Undetected	3311/44813 (7.39%)	3252/44852 (7.25%)	1.01 (0.93 to 1.10)*	1 more per 1000 (-2 to 5)	⊕⊕⊕O Moderate
Total cancer mortality (7)			No serious	No serious	No serious	No serious	Undetected	932/41091 (2.27%)	983/41116 (2.49%)	0.95 (0.87 to 1.04)*	1 fewer per 1000 (-3 to 1)	⊕⊕⊕⊕ High
All-cause mortality (8)			No serious	No serious	No serious	No serious	Undetected	2340/47361 (4.94%)	2433/47392 (5.13%)	0.96 (0.91 to 1.02)*	2 fewer per 1000 (-5 to 1)	⊕⊕⊕⊕ High
Major bleeding events	Dose of aspirin	≤ 100mg daily (16)	No serious	No serious	No serious	No serious	Undetected	1256/71279 (1.75%)	876/71455 (1.22%)	1.44 (1.32 to 1.57)#	5 more per 1000 (4 to 7)	⊕⊕⊕⊕ High
		100-300mg daily (2)	No serious	No serious	No serious	No serious	Undetected	54/11441 (0.47%)	34/11412 (0.30%)	1.58 (1.03 to 2.42)#	2 more per 1000 (0 to 3)	⊕⊕⊕⊕ High
		> 300mg daily(3)	No serious	No serious	No serious	No serious	Undetected	76/15215 (0.50%)	41/13116 (0.31%)	1.49 (1.02 to 2.18)#	2 more per 1000 (0 to 3)	⊕⊕⊕⊕ High
	Follow-up duration	1-5 years (12)	No serious	No serious	No serious	No serious	Undetected	801/58249 (1.38%)	529/58016 (9.11%)	1.51 (1.35 to 1.69)#	5 more per 1000 (3 to 6)	⊕⊕⊕⊕ High

		5-10 years (6)	No serious	No serious	No serious	No serious	Undetected	486/27602 (1.76%)	358/25917 (1.38%)	1.34 (1.17 to 1.53)#	4 more per 1000 (2 to 6)	⊕⊕⊕⊕ High
		>10 years (3)	No serious	No serious	No serious	No serious	Undetected	186/32230 (0.60%)	136/32253 (0.41%)	1.37 (1.17 to 1.81)	2 more per 1000 (1 to 3)	⊕⊕⊕⊕ High
Total bleeding events	Dose of aspirin	≤ 100mg daily (11)	No serious	No serious	No serious	No serious	Undetected	850/43659 (1.94%)	523/43744 (1.19%)	1.61 (1.37 to 1.89)*	13 more per 1000 (10 to 15)	⊕⊕⊕⊕ High
		100-300mg daily (4)	No serious	No serious	No serious	No serious	Undetected	3079/12719 (24%)	2306/12693 (18.17%)	1.45 (1.13 to 1.85)*	60 more per 1000 (50 to 70)	⊕⊕⊕⊕ High
		> 300mg daily(6)	No serious	Serious [e]	No serious	No serious	Undetected	331/8765 (3.78%)	123/5686 (2.16%)	1.72 (1.06 to 2.78)*	16 more per 1000 (10 to 22)	⊕⊕⊕O Moderate
	Follow-up duration	1-5 years (13)	No serious	No serious	No serious	No serious	Undetected	806/21374 (3.85%)	406/19618 (2.07%)	1.77 (1.57 to 1.99)#	15 more per 1000 (15 to 21)	⊕⊕⊕⊕ High
		5-10 years (4)	No serious	No serious	No serious	No serious	Undetected	3110/23919 (13.00%)	2332/19658 (11.86%)	1.33 (1.26 to 1.39)#	27 more per 1000 (20 to 33)	⊕⊕⊕⊕ High
		>10 years (2)	No serious	No serious	No serious	No serious	Undetected	207/21193 (0.98%)	158/21219 (0.74%)	1.32 (1.07 to 1.62)#	2 more per 1000 (1 to 4)	⊕⊕⊕⊕ High

Note: CVD: cardiovascular diseases; DM: diabetes mellitus; and CI: confidence interval. # fixed-effects model, * random-effects model.

[a] 4 trials, [b] 3 trials, [c] 1 trials, or [d] 2 trials were open-labelled and end-point blinded, most trials were low risk, the result had good robustness, and the evidence was not rated down; [e] heterogeneity occurred in them, the result had good robustness, and the evidence was rated down by one level.

Supplementary Table 3: Sensitivity analyses

Outcome	Double-blind, placebo- controlled studies [Study number; subject size; RR (95% CI); *P* value]	Subject size (≥ 2,000 in each group) [Study number; subject size; RR (95% CI); *P* value]	Studies published since the year 2000 [Study number; subject size; RR (95% CI); *P* value]	Excluding studies enrolling patients with increased risk of cancer [Study number; subject size; RR (95% CI); *P* value]
**Efficacy**				
Total cancer incidence	17 studies; N=152,747; 1.00(0.96 to 1.04); *P*=0.96	11 studies; N=156,636; 1.03 (0.99 to 1.07); *P*=0.10	14 studies; N=123,186; 1.01 (0.97 to 1.05); *P*=0.55	18 studies; N=176,818; 1.00 (0.97 to 1.04); *P*=0.31
Total cancer mortality	21 studies; N=145,470; 1.00 (0.93 to 1.07); *P*=0.95	9 studies; N=144,651; 1.03 (0.96 to 1.11); *P*=0.37	12 studies; N=103,498; 1.04 (0.96 to 1.12); P=0.39	23 studies; N=170,662; 1.00 (0.93 to 1.07); *P*=0.93
All-cause mortality	24 studies; N=163,661 0.99 (0.95 to 1.03); *P*=0.56	12 studies; N=165,432; 0.98 (0.94 to 1.02); *P*=0.39	13 studies; N=77,286; 0.99 (0.94 to 1.05); *P*=0.80	25 studies; N=187,732; 0.98 (0.94 to 1.02); *P*=0.31
**Safety**				
Major bleeding events	14 studies; N=145,692; 1.42(1.30 to 1.55); *P*<0.00001	10 studies; N=157,060; 1.41(1.29 to 1.54); *P*<0.00001	10 studies; N=114,077; 1.38 (1.25 to 1.51); *P*<0.00001	17 studies; N=171,208; 1.44(1.32 to 1.56); *P*<0.00001
Total bleeding events	17 studies; N=116,929; 1.35(1.31 to 1.40); *P*<0.00001	7 studies; N=108,002; 1.34(1.29 to 1.39); P<0.00001	8 studies; N=62,856; 1.41(1.20 to 1.65); *P*<0.0001	19 studies; N=128,160; 1.36(1.31 to 1.41); *P*<0.00001

RR, Risk Ratio, **CI**: confidence interval; **N,** the number of participants included in each analysis.

Supplemental references

1. Fowkes FG, Price JF, Stewart MC, et al. Aspirin for prevention of cardiovascular events in a general population screened for a low ankle brachial index: a randomized controlled trial. *JAMA.* 2010;303(9):841-848.
2. Baron JA, Cole BF, Sandler RS, et al. A randomized trial of aspirin to prevent colorectal adenomas. *N Engl J Med.* 2003;348(10):891-899.
3. A randomized, controlled trial of aspirin in persons recovered from myocardial infarction. *JAMA.* 1980;243(7):661-669.
4. Gaziano JM, Brotons C, Coppolecchia R, et al. Use of aspirin to reduce risk of initial vascular events in patients at moderate risk of cardiovascular disease (ARRIVE): a randomised, double-blind, placebo-controlled trial. *Lancet.* 2018;392(10152):1036-1046.
5. Group ASC, Bowman L, Mafham M, et al. Effects of Aspirin for Primary Prevention in Persons with Diabetes Mellitus. *N Engl J Med.* 2018;379(16):1529-1539.
6. Brighton TA, Eikelboom JW, Mann K, et al. Low-dose aspirin for preventing recurrent venous thromboembolism. *N Engl J Med.* 2012;367(21):1979-1987.
7. McNeil JJ, Nelson MR, Woods RL, et al. Effect of Aspirin on All-Cause Mortality in the Healthy Elderly. *N Engl J Med.* 2018;379(16):1519-1528.
8. Peto R, Gray R, Collins R, et al. Randomised trial of prophylactic daily aspirin in British male doctors. *Br Med J (Clin Res Ed).* 1988;296(6618):313-316.
9. Aspirin in coronary heart disease. The Coronary Drug Project Research Group. *Circulation.* 1980;62(6 Pt 2):V59-62.
10. Critical Leg Ischaemia Prevention Study G, Catalano M, Born G, Peto R. Prevention of serious vascular events by aspirin amongst patients with peripheral arterial disease: randomized, double-blind trial. *J Intern Med.* 2007;261(3):276-284.
11. Effect of aspirin alone and aspirin plus dipyridamole in early diabetic retinopathy. A multicenter randomized controlled clinical trial. The DAMAD Study Group. *Diabetes.* 1989;38(4):491-498.
12. Secondary prevention in non-rheumatic atrial fibrillation after transient ischaemic attack or minor stroke. EAFT (European Atrial Fibrillation Trial) Study Group. *Lancet.* 1993;342(8882):1255-1262.
13. Diener HC, Cunha L, Forbes C, Sivenius J, Smets P, Lowenthal A. European Stroke Prevention Study. 2. Dipyridamole and acetylsalicylic acid in the secondary prevention of stroke. *J Neurol Sci.* 1996;143(1-2):1-13.
14. Aspirin effects on mortality and morbidity in patients with diabetes mellitus. Early Treatment Diabetic Retinopathy Study report 14. ETDRS Investigators. *JAMA.* 1992;268(10):1292-1300.
15. Hansson L, Zanchetti A, Carruthers SG, et al. Effects of intensive blood-pressure lowering and low-dose aspirin in patients with hypertension: principal results of the Hypertension Optimal Treatment (HOT) randomised trial. HOT Study Group. *Lancet.* 1998;351(9118):1755-1762.
16. Ogawa H, Nakayama M, Morimoto T, et al. Low-dose aspirin for primary prevention of atherosclerotic events in patients with type 2 diabetes: a randomized controlled trial. *JAMA.* 2008;300(18):2134-2141.
17. Saito Y, Okada S, Ogawa H, et al. Low-Dose Aspirin for Primary Prevention of Cardiovascular Events in Patients With Type 2 Diabetes Mellitus: 10-Year Follow-Up of a Randomized Controlled Trial. *Circulation.* 2017;135(7):659-670.
18. Okada S, Morimoto T, Ogawa H, et al. Effect of Aspirin on Cancer Chemoprevention in Japanese Patients With Type 2 Diabetes: 10-Year Observational Follow-up of a Randomized Controlled Trial. *Diabetes Care.* 2018;41(8):1757-1764.
19. Yokoyama K, Ishizuka N, Uemura N, et al. Effects of daily aspirin on cancer incidence and mortality in the elderly Japanese. *Res Pract Thromb Haemost.* 2018;2(2):274-281.
20. Persantine and aspirin in coronary heart disease. The Persantine-Aspirin Reinfarction Study Research Group. *Circulation.* 1980;62(3):449-461.
21. Steering Committee of the Physicians' Health Study Research G. Final report on the aspirin component of the ongoing Physicians' Health Study. *N Engl J Med.* 1989;321(3):129-135.

22. Sturmer T, Glynn RJ, Lee IM, Manson JE, Buring JE, Hennekens CH. Aspirin use and colorectal cancer: post-trial follow-up data from the Physicians' Health Study. *Ann Intern Med.* 1998;128(9):713-720.
23. Belch J, MacCuish A, Campbell I, et al. The prevention of progression of arterial disease and diabetes (POPADAD) trial: factorial randomised placebo controlled trial of aspirin and antioxidants in patients with diabetes and asymptomatic peripheral arterial disease. *BMJ.* 2008;337:a1840.
24. de Gaetano G, Collaborative Group of the Primary Prevention P. Low-dose aspirin and vitamin E in people at cardiovascular risk: a randomised trial in general practice. Collaborative Group of the Primary Prevention Project. *Lancet.* 2001;357(9250):89-95.
25. Vidal AC, Howard LE, Moreira DM, Castro-Santamaria R, Andriole GL, Freedland SJ. Aspirin, NSAIDs, and risk of prostate cancer: results from the REDUCE study. *Clin Cancer Res.* 2015;21(4):756-762.
26. Swedish Aspirin Low-Dose Trial (SALT) of 75 mg aspirin as secondary prophylaxis after cerebrovascular ischaemic events. The SALT Collaborative Group. *Lancet.* 1991;338(8779):1345-1349.
27. Juul-Moller S, Edvardsson N, Jahnmatz B, Rosen A, Sorensen S, Omblus R. Double-blind trial of aspirin in primary prevention of myocardial infarction in patients with stable chronic angina pectoris. The Swedish Angina Pectoris Aspirin Trial (SAPAT) Group. *Lancet.* 1992;340(8833):1421-1425.
28. Hull MA, Sprange K, Hepburn T, et al. Eicosapentaenoic acid and aspirin, alone and in combination, for the prevention of colorectal adenomas (seAFOod Polyp Prevention trial): a multicentre, randomised, double-blind, placebo-controlled, 2 x 2 factorial trial. *Lancet.* 2018;392(10164):2583-2594.
29. Thrombosis prevention trial: randomised trial of low-intensity oral anticoagulation with warfarin and low-dose aspirin in the primary prevention of ischaemic heart disease in men at increased risk. The Medical Research Council's General Practice Research Framework. *Lancet.* 1998;351(9098):233-241.
30. Logan RF, Grainge MJ, Shepherd VC, Armitage NC, Muir KR, uk CAPTG. Aspirin and folic acid for the prevention of recurrent colorectal adenomas. *Gastroenterology.* 2008;134(1):29-38.
31. Farrell B, Godwin J, Richards S, Warlow C. The United Kingdom transient ischaemic attack (UK-TIA) aspirin trial: final results. *J Neurol Neurosurg Psychiatry.* 1991;54(12):1044-1054.
32. Cook NR, Lee IM, Gaziano JM, et al. Low-dose aspirin in the primary prevention of cancer: the Women's Health Study: a randomized controlled trial. *JAMA.* 2005;294(1):47-55.
